# Quantitative MRI Pharmacokinetic Modeling and MALDI Mass Spectrometry Imaging Biodistribution of a pH Mapping MRI Contrast Agent

**DOI:** 10.7150/thno.128499

**Published:** 2026-07-05

**Authors:** Zinia Mohanta, Caitlin M. Tressler, Sadakatali Gori, Aruna Singh, Julia Stabinska, Shaowei Bo, Sana Karbalaei, Sophie Sall, Assaf Gilad, Hernando Lopez Bertoni, Eric Gale, Kristine Glunde, Michael T. McMahon

**Affiliations:** 1 Russell H. Morgan Department of Radiology and Radiological Science, Johns Hopkins School of Medicine, Baltimore, Maryland 21205, United States of America.; 2 F.M. Kirby Research Center for Functional Brain Imaging, Kennedy Krieger Institute, Baltimore, Maryland, 21205, United States of America.; 3 The Johns Hopkins University Applied Imaging Mass Spectrometry Core and Service Center, Division of Cancer Imaging Research, The Russell H. Morgan Department of Radiology and Radiological Science, The Johns Hopkins University School of Medicine, Baltimore, Maryland, 21205, United States of America.; 4 The Sidney Kimmel Comprehensive Cancer Center, The Johns Hopkins University School of Medicine, Baltimore, Maryland, 21205, United States of America.; 5 Center for Translational Pharmacology, Department of Pharmacy and Pharmaceutical Sciences, St. Jude Children’s Research Hospital, Memphis, Tennessee, 38105, United States of America.; 6 Department of Medical Imaging, The Affiliated Guangdong Second Provincial General Hospital of Jinan University, Guangzhou, 510317, China.; 7 Athinoula A. Martinos Center, Department of Radiology, Massachusetts General Hospital and Harvard Medical School, Boston, Massachusetts, 02129, United States of America.; 8 Department of NeuroOncology, Kennedy Krieger Institute, Baltimore, Maryland, 21205, United States of America.; 9 Department of Neurology, Johns Hopkins School of Medicine, Baltimore, Maryland, 21205, United States of America.; 10 Scojen Institute of Synthetic Biology, Reichman University, Herzliya, 4610101, Israel.

**Keywords:** pH sensor, Imidazole-based probe, MALDI imaging, renal biodistribution, non-invasive molecular imaging

## Abstract

Chemical exchange saturation transfer (CEST) MRI enables noninvasive mapping of tissue pH, a key biomarker of renal physiology and disease. We report a streamlined, three-step synthesis of diGlu, an anionic imidazole-4,5-dicarboxamide bearing two glutamate substituents, obtained at ≥99.5% purity using SOCl_2_/DMF activation, low-temperature amidation, and mild HCl deprotection-avoiding phenyl-ester intermediates and TFA. The molecule presents two exchangeable protons at 7.7 ppm (imidazole) and 4.5 ppm (amide) whose pH-dependent exchange rates enable a two-offset ratiometric, concentration-independent readout.

**Methods:**

C57BL/6J mice received tail-vein injections of 250, 500, or 750 mg kg^-1^ I45DC-diGlu for our imaging studies. We established a novel biodistribution and pharmacokinetic analysis methodology employing matrix-assisted laser desorption/ionization (MALDI) mass spectrometry imaging (MSI) for biodistribution, CEST imaging and region of interest (ROI)-wise pharmacokinetic (PK) modeling, fitting SE (%) time-courses with log-normal (LN) and Uptake-Plateau-Decay (UPD) models and selecting the model by AICc on the SE time course curves.

**Results:**

MALDI-MSI confirmed selective renal localization and unmetabolized probe in urine, with negligible liver or pancreas accumulation. The LN model was best for fitting the CEST MRI data at low doses (unimodal kinetics), whereas UPD captured rise-hold-washout at 750 mg kg^-1^, yielding interpretable biomarkers (SE_max_, TTP, AUC_total_, t_1/2_). Robust renal CEST contrast was observed at ≥ 500 mg kg^-1^. Voxel-wise pH maps reproduced the expected corticomedullary gradient (mean ± SD): cortex 6.86 ± 0.087, outer medulla 6.80 ± 0.073, inner medulla 6.61 ± 0.079, with pH estimates largely dose-independent.

**Conclusions:**

Together, the iodine- and metal-free chemistry, dual-offset window, dose-robust pH mapping, and MALDI-validated biodistribution position I45DC-diGlu as a practical scaffold for quantitative renal pH imaging and as a foundation for translational studies for renal pathophysiology.

## Introduction

Non-invasive imaging of tissue pH has emerged as a powerful biomarker due to the tight control of the acid-base balance in the human body, with alterations in pH associated with pathological changes in the kidneys, increased aggression of tumors, and death of cells after transplantation.^1^-^4^ Gillies and colleagues highlighted the biological importance of acidic tumor microenvironments and their role in cancer progression and resistance to therapy,^5^, ^6^ and we and others have discovered that alterations in kidney pH can be linked to progression in acute kidney injuries or kidney disease.^7^-^10^ A number of advanced pH imaging probes have been developed over multiple imaging modalities, including fluorescent pH-sensitive dyes^11^-^13^, electron paramagnetic resonance detectable pH-sensitive nitroxyl radical probes,^14^ photoacoustic imaging (PAI) probes,^15^ pH (low) insertion peptide (pHLIP) probes for positron emission tomography (PET) 16 and several types of magnetic resonance probes including hyperpolarized probes,^17^, ^18^ T_1_ relaxation probes^19^ and chemical exchange saturation transfer (CEST) probes. Indeed, pHLIP-iCG fluorescent probes and iopamidol CEST MRI probes are now being evaluated for characterizing tumor and kidney pH in patients,^20^, ^21^ which should further spur interest. With the recent establishment of pH as a biomarker for kidney function in a number of preclinical models, it is time to migrate testing of pH probes in larger animal models to enable translation.

Chemical exchange saturation transfer (CEST) MRI is an emerging non-invasive imaging technology due to its ability to detect low-concentration solutes, detection in deep tissue and multi-frequency detection enabling ratiometric imaging and making this technology uniquely suited for measuring the pH of tissue.^22^-^24^ One of the general issues with CEST MRI pH mapping is that the probes have to be administered at high concentrations to enable not just measuring the signal from one type of labile proton, but accurately measuring the ratio of two different proton signals. Triiodobenzenes including iopamidol are suitable as diamagnetic CEST (diaCEST) MRI pH probes due to their very high biocompatibility and strong contrast provided by aromatically conjugated N-H protons.^25^ One challenge of using these triiodobenzenes for pH mapping is that the protons are still relatively close to background amide proton contrast and to water, restricting the saturation field strengths that can be employed which ultimately impacts sensitivity. The intramolecular hydrogen bonded agents are a high-performance class of CEST probes with large labile proton chemical shifts (> 5.0 ppm) and well-tuned proton exchange rates. Notable examples include the salicylates, anthranilates, porphyrins, hydrazones, acetanilids and imidazoles.^26^-^31^ Of the intramolecular hydrogen bonded agents, the imidazole 4,5-dicarboxamide (I45DC) scaffold is particularly powerful due to the large chemical shifts of two labile protons with favorable exchange rates enabling ratiometric pH imaging in the physiological range.^32^ Subsequent efforts by Bo *et al.* introduced a number of I45DC derivatives and demonstrated that I45DC-diGlu (diGlu) performed particularly well and can visualize changes in kidney pH following unilateral ureteral obstruction (UUO).^33^ With that said, prior preparations have relied on phenyl ester intermediates, extended refluxing, and trifluoroacetic acid-mediated deprotection, which result in modest yields (∼58-66%), increased synthetic complexity, and limited scalability for translational development. Furthermore, the *in vivo* biodistribution, pharmacokinetics (PK) and metabolic stability of diGlu and dose dependence of the pH maps have not been fully characterized and defined.

**Figure 1:**
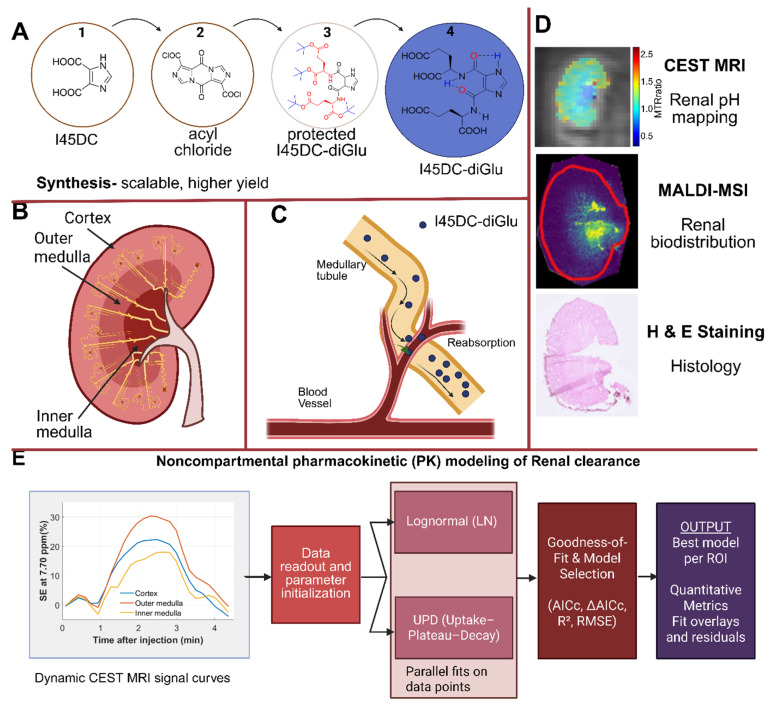
From bench to kidney: overview of I45DC-diGlu synthesis, renal biology, and multimodal read-outs.** (A)** Three-step scalable route (I45DC → acyl chloride → protected intermediate → deprotected product) affords I45DC-diGlu with 85% yield in final step. **(B)** Murine kidney illustration- cortical, outer-medullary, and inner-medullary zones that display characteristic pH gradients.** (C)** Schematic of first-pass renal handling: freely filtered I45DC-diGlu (blue dots) traverses medullary tubules, with minimal re-absorption, and is rapidly cleared into urine.** (D)** Multimodal validation. Top: CEST MRI pH map (rainbow scale) demonstrates corticomedullary gradient. Middle: MALDI-MSI ion map confirms medullary accumulation of [diGlu+H]^+^ /Na⁺/K⁺ adducts. Bottom: H&E section shows preserved histology post-injection. **(E)** Block diagram of PK modeling for dynamic renal CEST MRI: For each ROI, ROI curves were extracted, used for data readout and parameter initialization, parallel log-normal and UPD fits performed on the ROI data points, AICc-based model selection applied, and then quantitative metrics and residual diagnostics were derived. Created in https://BioRender.com.

In this study, we report a re-engineered synthesis and novel simplified protocol to characterize the organ biodistribution, PK and dose dependence renal pH maps. This protocol involves collecting MALDI images at one time point and serial CEST MRI scans of unlabeled diGlu instead of using radiolabeled drug and gamma counting/scintigraphy or liquid chromatography-mass spectrometry for measuring biodistribution or serial blood and urine collection for characterizing PK. **Figure 1** provides a visual overview of our study design. **Figure 1A** summarizes our streamlined three-step, high-yield synthesis of diGlu which avoids phenyl-ester intermediates and TFA deprotection and improved product purity. We performed dose escalation studies in mice for the kidney mapping and clearance with **Figure 1B** highlighting the renal regions of interest- cortex, outer and inner medulla. **Figure 1C** illustrates first-pass renal handling, with intravenously delivered diGlu freely filtered into the medullary tubules and rapidly cleared in urine. **Figure 1D** displays our novel, multimodal drug distribution imaging and PK modeling pipeline: MALDI-MSI for biodistribution, dual-offset CEST MRI for characterizing kidney PK and displaying the dose dependence of pH mapping and Hematoxylin and Eosin (H&E) histology for characterizing tissue integrity. This novel characterization method could be applied to characterize the biodistribution and pharmacokinetics for other imaging agents as well. To quantify renal handling of diGlu from the dynamic CEST curves, we implemented a compact, data-driven pharmacokinetic framework (**Figure 1E**). This yielded interpretable metrics (maximum signal enhancement, SE_max_, total area under curve, AUC_total_, half-life time period after injection, t_1/2_) that we use throughout to summarize renal uptake and clearance. Finally, we show dose-dependent kinetics that inform protocol selection and establish how to apply the diGlu probe for use in larger animal models.

## Materials and Methods

### Reagents and materials

4,5-Imidazoledicarboxylic acid, tetrahydrofuran (THF), benzene, and thionyl chloride were purchased from Millipore Sigma (Burlington, MA, USA). L-Glutamic acid di-tert-butyl ester hydrochloride was obtained from Chem-Impex (Wood Dale, IL, USA). N,N-Dimethylformamide (DMF), N,N-diisopropylethylamine (DIPEA), petroleum ether (ACS grade), Optima^TM^ HPLC-grade solvents, and silica gel (60 Å, 0.030-0.200 mm) were purchased from Thermo Fisher Scientific (Waltham, MA, USA). Ethyl acetate (≥ 99.7%), acetone, acetonitrile (HPLC grade, ≥ 99.9%), trifluoroacetic acid (99%), dichloromethane (anhydrous, ≥ 99.8%), and HPLC-grade water were purchased from Sigma-Aldrich (Burlington, MA, USA).

Thin-layer chromatography (TLC) was performed on pre-coated silica gel 60 F254 plates (200 µm thickness, 20 × 20 cm). Flash column chromatography was performed using silica gel 60 Å (0.030-0.200 mm). Rotary evaporation was performed under reduced pressure using a vacuum aspirator. Preparative HPLC was performed on a Shimadzu Premier C18 column (250 × 20 mm, 5 µm) at a flow rate of 5 mL/min using a water-acetonitrile gradient.

Unless otherwise stated, NMR spectra were recorded on a 750 MHz spectrometer. Chemical shifts (δ) are reported in ppm relative to residual water in D_2_O.

### Synthesis of diGlu

**Figure 2:**
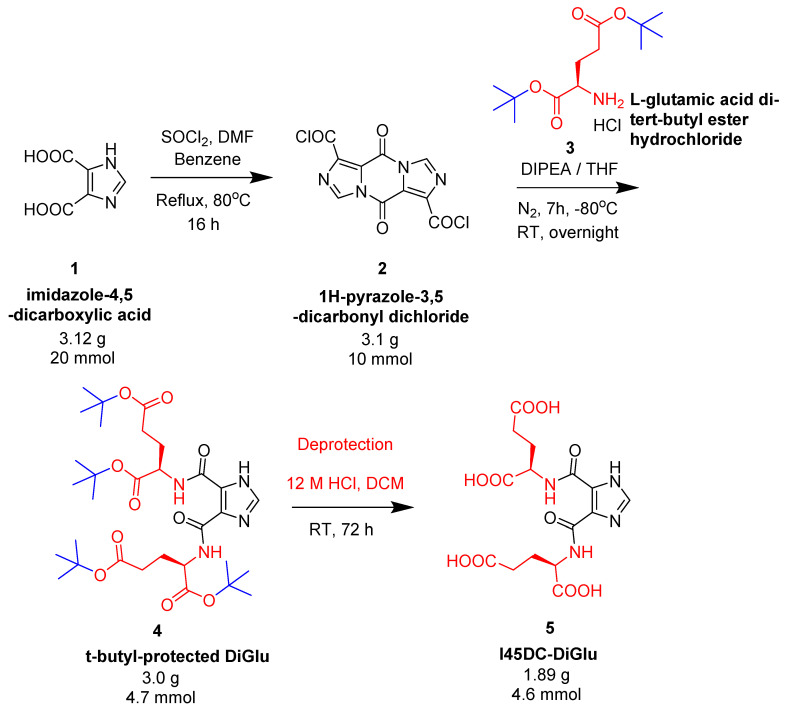
** Improved synthesis scheme for diGlu**. Step 1: Conversion of imidazole-4,5-dicarboxylic acid (1) to imidazole-4,5-dicarbonyl dichloride (2) with SOCl_2_ (cat. DMF); Step 2: Bis-acylation of 2 with *L*-glutamic acid di-tert-butyl ester · HCl (3) to give the protected di-glutamyl intermediate (4); Step 3: Global deprotection with 12 M HCl (aq.), to afford diGlu (5, free acid) with red fragments denoting the *L*-glutamyl arms; blue groups denoting *tert*-butyl protecting groups.

The reaction scheme for preparing diGlu is shown in **Figure 2** with the steps described in detail below.

**Step 1: Formation of Pyrazole-3,5-dicarbonyl dichloride (Intermediate 2).** Imidazole-4,5-dicarboxylic acid (1) (3.12 g, 20 mmol) was dissolved in benzene (30 mL), followed by dropwise addition of thionyl chloride (13 mL, 0.2 mol) with stirring at room temperature. Catalytic DMF (0.77 mL, 10 mmol) was added, and the mixture was refluxed at 80 °C for 16 h. After cooling to room temperature, the crude product was isolated by vacuum filtration to yield 3.1 g of 1H-pyrazole-3,5-dicarbonyl dichloride** (2)** as a yellowish solid.

**Step 2: Coupling with Protected Glutamic Acid.** Compound 2 (3.1 g, 10 mmol) was added to a nitrogen-purged round-bottom flask and suspended in THF (50 mL). The suspension was cooled to -80 °C using a dry ice/acetone bath. Separately, L-glutamic acid di-tert-butyl ester hydrochloride (3) (12 g, 45 mmol) was dissolved in THF (50 mL) and added via cannula to the suspension with stirring. After complete addition, the mixture was stirred for 2 h at -80 °C, followed by dropwise addition of DIPEA (14 mL). The mixture was stirred for 5 h at -80 °C, allowed to gradually reach room temperature, and stirred overnight. The product was collected by vacuum filtration.

The organic phase was extracted with ethyl acetate (30 mL), washed sequentially with saturated sodium bicarbonate (3×) and brine (3×), and dried. The solvent was evaporated under reduced pressure to obtain 4.6 g of crude t-butyl-protected diGlu **(4)**.

**Purification of Compound 4.** Compound 4 was purified by column chromatography on silica gel using a petroleum ether/ethyl acetate (PE/EtOAc) gradient: starting from 8:1 to 5:1, 3:1, 1:1, and finally to 100% EtOAc. Fractions were monitored via TLC, and the product-containing fractions (R_f_ = 0.72; petroleum ether:ethylacetate 1:2) were combined and dried to yield 3.4 g of purified compound 4 (73.9% yield).

**Step 3: Deprotection to Yield I45DC-diGlu.** Compound 4 (3.0 g, 4.6 mmol) was dissolved in dichloromethane (30 mL), and aqueous 12 M HCl (30 mL) was added to form a biphasic mixture. The mixture was stirred at room temperature for 72 h. The aqueous layer was separated and concentrated under vacuum to yield a white residue, which was redissolved in water (30 mL) and freeze-dried to afford I45DC-diGlu (1.89 g, 4.5 mmol, 90% yield).

Purification was then performed using gradient silica gel flash chromatography (PE/EtOAc 8:1→0:1). Final purification was performed by preparative C18 HPLC (MeCN/H_2_O 0-30 % in 30 min) and lyophilization to afford 1.65 g of I45DC-diGlu as a white powder (85% recovery after purification).

The identity and purity of I45DC-diGlu were confirmed by orthogonal analytics. Analytical HPLC (C18, H_2_O/MeCN gradient) showed a single peak with ≥ 99.5% area. HRMS (MALDI-TOF) matched the calculated exact masses for [M+H]⁺, [M+Na]⁺, and [M+K]⁺. ^1^H NMR (750MHz, D₂O) was consistent with the expected structure (δ 7.7, imidazole-H; δ 4.5, α-CH; δ 2.38/2.18-1.92, β/γ-CH, **Figure S1A**).

- **¹H NMR** (750 MHz, D_2_O): δ 7.7 (s, 1H), 4.5 (dd, J = 6.9, 3.9 Hz, 2H), 2.38 (t, J = 5.4 Hz, 4H), 2.18-2.09 (m, 2H), 2.00-1.92 (m, 2H).
- **HPLC** (MeCN/H_2_O 10:90, 5 mL/min): 25 min
- **MALDI-TOF** (m/z 415.1102 [M+H]^+^, 437.0922 [M+Na]^+^, 453.0655 [M+K]^+^).

An HPLC chromatogram of the final injected I45DC-diGlu compound confirms sample purity (**Figure S1B**).

### Phantom studies and pH calibration

To establish the pH sensitivity of I45DC-diGlu, *in vitro* CEST MRI measurements were performed on phantoms containing the contrast agent at various pH values. Solutions of 40 mM I45DC-diGlu were prepared in 1× PBS (10 mM phosphate, 137 mM NaCl, 2.7 mM KCl) and titrated to pH values between 5.7 and 7.1 using small aliquots of NaOH or HCl. The pH of each solution was measured using a microelectrode (Orion Single Junction Thin Stem Glass Bodied Combination pH Electrode, 9103BNWP, Orion Star A111 pH meter, Thermo Fisher Scientific) calibrated at room temperature. Samples (300 µL) were loaded into 5 mm NMR tubes and positioned in the holder. MRI experiments were performed on a 11.7 T vertical Bruker BioSpec scanner. CEST imaging was conducted using a single-slice RARE readout (RARE factor = 16, matrix size = 64 × 64, field of view = 20 × 20 mm^2^, slice thickness = 1.5 mm). A continuous wave saturation pulse was applied with saturation time = 2 s, saturation B_1_ = 5 µT as image preparation. Z-spectra were acquired by incrementing the frequency offset from -12 to +12 ppm in 0.2 ppm steps.

### Z-spectrum


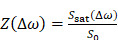

(1)

where S_sat_(Δω) is the signal with saturation at frequency offset Δω (also S_z_) and S_0_ is the unsaturated reference signal (0 ppm or no-sat).

**Magnetization transfer ratio (MTR).** The saturation transfer effect at each offset was expressed as


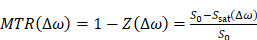

(2)

### Asymmetric magnetization transfer ratio


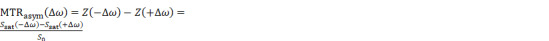

(3)


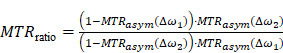

(4)

Ratiometric pH mapping using the pH calibration curve was employed to eliminate concentration dependence and enable accurate, quantitative pH estimation.

A pH calibration curve was generated by plotting the ratio of CEST signal at 4.5 ppm to that at 7.7 ppm across the known pH range. The resulting calibration curve was fit using a third order polynomial function


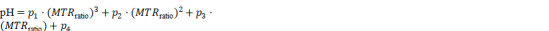

(5)

The values of the coefficients, p_1_, p_2_, p_3_ and p_4_ were used in the equation applied to *in vivo* datasets for pixel-wise pH map generation.

### Simulation of ratiometric pH response under preclinical and clinical-field conditions

Numerical simulations were carried out using a three-pool Bloch-McConnell model that included two exchanging solute pools, corresponding to the I45DC-diGlu resonances at 7.7 and 4.5 ppm, along with a water pool. Z-spectra were simulated across an offset range from -6000 to 6000 Hz with 50 Hz increments. For the preclinical condition, the simulations used a proton Larmor frequency of 500 MHz, corresponding to 11.7 T, with a saturation power (B_1_) = 5 μT and a saturation duration, T_sat_ = 2 s. The simulations were run over a pH range of 5.7 to 7.1, and the exchange rates for the 7.7 and 4.5 ppm pools were adjusted as a function of pH using values interpolated from predefined lookup tables based on our previous measurements.^32^

k_7.7_ = [35000 14250 6200 4750 4050 3950];

k_4.5_ = [4500 6000 8000 13000 14000 14500];

The total solute fraction was set to correspond to a concentration of 40 mM, with greater relative weighting assigned to the 7.7 ppm pool. Water T_1_ and T_2_ values in the ranges of 2.5-3.0 s and 2.0-2.5 s, respectively, were used. From the simulated Z-spectra, MTR_asym_ curves were calculated and then smoothed with a Savitzky-Golay filter. Saturation transfer amplitudes at 4.5 and 7.7 ppm were extracted using ST = 1 - Z, and the ratiometric metric was defined as ST_7.7_/ST_4.5_. A third-order polynomial was then fit to describe the relationship between the ratiometric metric and pH, and the fitting error was quantified using the root-mean-square error (RMSE).

### Animal preparation and MRI acquisition

All procedures were approved by the institutional IACUC. All *in vivo* imaging experiments were performed on an 11.7 T Bruker BioSpec horizontal bore animal scanner (Bruker Biospin GmbH, Ettlingen, Germany) equipped with an 8-channel phased-array mouse body coil. Male C57BL/6J mice (3-4 months old) were anesthetized with 1.5-2.0% isoflurane in oxygen and placed on a heated imaging cradle to maintain body temperature at 37 °C. The respiratory rate was monitored continuously throughout the scan and maintained at 60-100 breaths/min.

A high-resolution T_2_-weighted anatomical scan was acquired using a multi-slice RARE sequence (TE/TR = 20/4000 ms, matrix size = 128 × 128, slice thickness = 1.5 mm, RARE factor = 8). A single 1.5 mm coronal slice encompassing both kidneys was selected for dynamic CEST imaging.

**B_0_ correction**. WAter Saturation Shift Referencing (WASSR) protocol was performed using 42 frequency offsets between -1.5 and +1.5 ppm, with 10 rectangular saturation pulses (300 ms each, inter-pulse delay = 10 μs, saturation power, B_1_ = 1.2 μT).

**Dynamic CEST.** Time**-**series imaging was conducted using a single-shot RARE sequence with centric encoding. Saturation was obtained using continuous RF pulse of 2000 ms (TE/TR = 3.5/5000 ms, B_1_ = 5.0 μT, total saturation time, T_sat_ = 2 s). Images were acquired repeatedly at 4.5 ppm and 7.7 ppm offsets (interleaved). A total of 180 time points were captured over ~30 mins. Pre-injection baseline (M_0_) images were collected, followed by intravenous injections of I45DC-diGlu at 250 - 750 mg kg^-1^ via tail vein, and post-injection acquisition began immediately. The temporal resolution was ~10 s per time point. Imaging was performed after iopamidol injection under identical experimental conditions and offsets used were 4.2 and 5.5 ppm. **Figure S10B** shows schematic timing diagram of the CEST pulse sequence, including the saturation period, recovery interval, and readout module.

**Cohorts.** 2 groups of 5 mice were injected with 250 mg kg^-1^ and 500 mg kg^-1^ of diGlu and 3 mice were injected with 750 mg kg^-1^ of diGlu. 1 group of 3 mice were injected with 1.5 g iodine kg^-1^ of iopamidol.

### CEST MRI analysis

All processing was performed in MATLAB (R2021b) with in-house scripts. Data were first normalized, B_0_-corrected, and then used to derive quantitative maps of magnetization transfer ratio (MTR), ΔMTR, ratiometric MTR, and pH.

**B₀ correction (WASSR-based)**. To correct for magnetic field inhomogeneities, a separate water saturation shift referencing (WASSR) acquisition was performed with low-power saturation centered around the water resonance. For each voxel, the WASSR Z-spectrum was interpolated and the frequency corresponding to the minimum signal was identified, yielding the voxel-specific frequency shift dB_0_. A B_0_ map in parts per million (ppm) was generated, and CEST data were corrected by re-centering the frequency axis to water. Voxels with |dB_0_| exceeding a predefined tolerance of 0.3 ppm were excluded from further analysis.

**Normalization and Z-spectrum.** For each voxel, the signal acquired after saturation at frequency offset Δω (denoted S_sat_) was normalized to the unsaturated reference (S_0_) to generate the Z-spectrum (same as Equation 1). While WASSR Z-spectrum was acquired to estimate the voxel-wise B_0_ map for frequency correction, we did not sample a full Z-spectrum for dynamic acquisition. Instead, we used a dual-offset window, acquiring two CEST-saturated images at predefined offsets (4.5 and 7.7 ppm) at each time point for the ratiometric readout with the frequencies adjusted to be correct based on the WASSR B_0_ map. For iopamidol, offsets 4.2 and 5.5 ppm were used.

**Magnetization transfer ratio (MTR).** MTR was calculated using Equation (2).

**Dynamic change in saturation (ΔMTR).** To assess dynamic changes after agent administration, pre- and post-injection windows were defined from the dynamic time series. For each offset, ΔMTR was computed as:


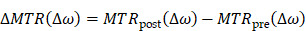

(6)

**Ratiometric analysis.** A ratiometric MTR metric was calculated using ΔMTR values at two CEST offsets (Δω_1_, Δω_2_), which reduces confounding by non-specific effects and B_0_/B_1_ variations:


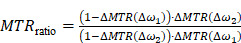

(7)

**pH calibration.** Voxel-wise pH values were obtained by converting MTR_ratio_ into pH using the cubic calibration curve derived from independent phantom experiments (Equation 5). The final pH maps were restricted to the physiologic range of 5-8.

**Parametric maps.** We computed (i) MTR_post_ at 7.70 and 4.50 ppm, (ii) ΔMTR = MTR_post_ - MTR_pre_ at both offsets, (iii) 
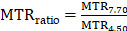
, and (iv) pH maps using the phantom-derived ratiometric calibration (MTR_4.5_/MTR_7.7_ → pH, B_1_ = 5 µT).

**ROIs.** Cortex (C), outer medulla (OM), and inner medulla (IM) were delineated along the cortico-papillary axis on the reference image and propagated to all maps (**Figure 1B**). ROI quality was reviewed by two raters. For whole-kidney quantitative analysis, regions corresponding to the cortex and outer medulla were included, while the inner medulla was excluded due to this region’s reduced signal-to-noise ratio. For renal compartment-wise analysis, however, all three regions (cortex, outer medulla, and inner medulla) were included.

**Time courses.** For each ROI at 7.70 ppm and 4.50 ppm, SE (%) time-courses were extracted, lightly smoothed (moving average, window = 3 points), and analyzed with time referenced to the post-injection dynamic acquisition start.

Signal-enhancement time courses were computed as


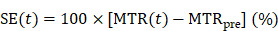

(8)

These SE (%) time courses at 7.70 ppm were used for pharmacokinetic (PK) modeling.

### Noncompartmental PK modeling of renal clearance

In renal DCE-MRI and CEUS, researchers routinely quantify wash-in, plateau, and wash-out through time-intensity-curve (TIC) features and semi-quantitative models (e.g., time-to-peak, wash-in slope, peak intensity, AUC, wash-out rate), validating the utility of a three-phase description when an explicit AIF is unavailable. 34 Building on these precedents and the renal CEST urography literature emphasizing dynamic pH imaging, we introduced a CEST-specific, phenomenological Uptake-Plateau-Decay (UPD) function to explicitly encode a transient hold between rise and wash-out, alongside a log-normal (LN) alternative for unimodal curves.^35^-^40^ Several candidate kinetic models (including gamma-variate and Weibull) were initially evaluated, and Log-normal (LN) and Uptake-Plateau-Decay (UPD) were selected for primary analysis based on curve-shape suitability and AICc performance. We compared the two candidate models, a log-normal (LN) and a phenomenological UPD model and selected the preferred description by AICc. From the preferred model we derived compact biomarkers: SE_max_, TTP, total AUC, t_1/2_ (half-life). This approach mirrors dynamic CEST curve-shape practice and non-CEST renal kinetics while remaining physiology-agnostic and reproducible. It is intended as a bridge toward future AIF-based compartment modeling. Implementation details (constraints, fitting, and GOF metrics) follow.

### Data and regions of interest

Dynamic CEST MRI time-series were exported from MATLAB figures containing ROI-wise signal-enhancement (SE) versus time. For each subject, three renal regions were analyzed (inner medulla, outer medulla, cortex) at multiple doses. SE was expressed as percentage change relative to the pre-injection baseline; if curves were fractional, they were scaled to %.

### Candidate models

We evaluated fitting this data to two parametric models that capture common bolus shapes.

- Log-normal (LN)

This has been applied to modeling iodinated contrast plasma clearance previously as measured through serial blood collection 39 and employs the expression:


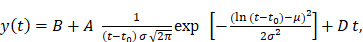

(9)

with baseline B, amplitude A > 0, log-time location µ, log-scale σ > 0, bolus onset t_0_, and an optional linear drift term D.

- Uptake-Plateau-Decay (UPD) model

We developed the UPD model as a piecewise exponential linear hybrid model to model the dynamic CEST signal-enhancement time-courses (SE, %) with a phenomenological UPD function that captures the observed rise-hold-washout shape without assuming a specific compartment physiology. Let *t* be time (min) and 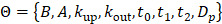. The model is:


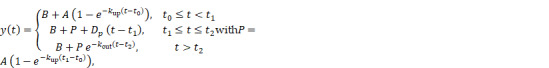

(10)

where, *B* is the baseline SE; *A* is the uptake amplitude; *k_up_* and *k_out_* (min^-1^) are the uptake and washout rates (tail half-life 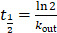); t_0_ is the bolus-arrival time; 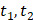
are the plateau bounds; 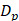
is the plateau drift (%·min^-1^). We enforced *k_up_*, *k_out_* > 0 and a minimal plateau duration t_2_ ≥ t_1_ + ɛ (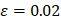
min); |D_p_| was softly capped (≤ 3 %·min^-1^) to avoid overfitting tiny wiggles. If no clear plateau was present, the model collapsed to a tiny window at the peak (t_1_ = t_max,_ t_2_ = t_1_ + ɛ) for continuity.

### Fitting and model selection

Nonlinear least squares with robust bisquare weights (MATLAB nlinfit) was used when available; otherwise, Nelder-Mead (fminsearch) minimized the residual sum of squares. Goodness metrics included:

- corrected Akaike information criterion AICc,
- coefficient of determination R^2^,
- root-mean-square error (RMSE).

The preferred model per curve was the one with minimum AICc as described previously 41, 42. We report ΔAICc for both models relative to the best.

AICc was computed as


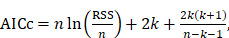

(11)

where *n* is the number of data points, *k*, the number of free parameters, and 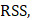
the residual sum of squares from the nonlinear least-squares fit. The model with the lowest AICc was selected as preferred; we also report ΔAICc = AICc_model_ - AICc_best_, with ΔAICc > 2, interpreted as meaningfully worse model. Parameter half-lives and post-peak AUCs are reported from the preferred model.

### Derived kinetic metrics

All metrics used the data points from the SE curves for each ROI. With 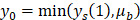
as baseline reference:

- Peak enhancement SE_max_ and time-to-peak (TTP)
- Areas under the curve (AUC, trapezoidal)

The area under the curve (AUC) for post-peak signal intensity versus time was calculated using the trapezoidal rule to quantify overall exposure of I45DC-diGlu within the renal ROI.


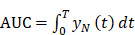
(reported in minutes and %·min). (12)

Here, 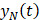
denotes the normalized percent signal change relative to pre-injection baseline, and 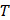
corresponds to the total post-injection acquisition time. The resulting AUC, expressed in %·min, represents the cumulative contrast enhancement over time.

- Half-life (t_1/2_)

The half-life (in minutes) is the time at which the signal on the decay limb first falls to 50% of the peak value from the time of injection.

### Diagnostics and visualization

Residuals (data-fit) were inspected versus time and via PDF-normalized histograms. Plots displayed data, optional helper curves (clearly labeled “interp/smooth”), and both model fits with legends showing ΔAICc. X/Y limits were standardized across panels to aid visual comparison.

Residual frequency distributions were examined to assess central tendency and dispersion, and the temporal dependence of residuals was evaluated using the autocorrelation function (ACF). The lag-1 autocorrelation coefficient (ACF_1_) was computed as a summary measure of short-range temporal correlation. Approximate confidence bounds for the ACF were estimated assuming white-noise residuals. These analyses were performed on the original sampled data without outlier removal and were used to assess residual structure, temporal dependence, and overall model stability.

### Outputs

For each ROI, we stored the raw series, preprocessing estimates, fit parameters, GOF statistics, kinetic metrics, residual diagnostics, and the AICc-based model choice (“Log-normal” or “UPD (piecewise)”). Aggregate tables (CSV) and figures (fits, residuals, histograms) were generated for reporting and reproducibility.

### MALDI imaging and histological assessment

DiGlu (500 mg kg^-1^) was injected through the tail vein. Animals were euthanized immediately with carbon dioxide for three minutes followed by cervical dislocation. Kidney, liver, and pancreas were harvested and frozen on liquid nitrogen vapors. Tissues were stored at -80 °C until cryo-sectioning. Tissues were cryo-sectioned onto cold, washed, and dried indium tin oxide (ITO) slides (Delta Technologies, Loveland, CO) on a Leica cryostat (Leica, Wetzlar, Germany) at 10-micron thickness. All kidney, liver and pancreas tissue sections were cryo-sectioned at the same tissue depth for each specific organ covering the same tissue regions per organ. Slides were warmed to room temperature in a vacuum desiccator prior to matrix application. 2,5-dihydroxybenzoic acid (DHB) was sublimated using an HTX SubliMate (HTX Technologies, LLC, Chapel Hill, NC). Thirty milligrams of DHB were dissolved in 1.5 mL of acetone and pipetted in a triangular pattern onto the SubliMate wafer. Slides were chilled to -10 °C inside the sublimation chamber prior to sublimation. Once dried, the matrix was sublimated at 40 mTorr for 5 minutes at 200 °C. Slides were warmed to room temperature under vacuum after sublimation.

Urine from mice was collected by placing them on 96 well-plates after the effect of anesthesia wore off. Urine from control mice and mice injected with 250 and 750 mg kg^-1^ of diGlu were collected. Target plate experiments were performed with pure diGlu or 1 ml of mouse urine spotted onto a ground steel 396 well target plate with saturated DHB.

MALDI mass spectrometry imaging was performed on a Bruker timsToF FleX MALDI-2 (Bruker Daltonics, Billerica, MA). Experiments were performed in positive ion mode with 500 laser shots per pixel and a 50-micron single beam laser. A 50-micron raster was used. The laser was set to 90% intensity and an m/z range of 85 to 1,000 Da was collected. Data was imported into SCiLS lab (Bruker Daltonics, Billerica, MA) and normalized to Total Ion Count (TIC) for analysis. Pixel intensity plots and box-and-whisker plots were generated for individual tissue sections from all tissues in SCiLS lab. Receiver Operating Characteristic (ROC) curves and ROC area under the curve (AUC) were generated to test if control *versus* diGlu treated tissues contained significantly different diGlu signals. AUC values greater than 0.750 or smaller than 0.250 were considered significantly different. All MALDI imaging pixels per organ and treatment group were pooled for the ROC analyses. Since all kidney, liver and pancreas tissue sections were sectioned at the same tissue depth covering the same tissue regions for each organ, overall ion suppression across all tissue pixels per organ was identical in the quantified MALDI imaging pixels of control kidney, liver or pancreas *versus* kidney, livers, or pancreas from diGlu treated animals. Therefore, quantification of diGlu ion adducts directly reports on differences in diGlu tissue concentration.

After MALDI imaging, the matrix was removed by submerging the slides in 100% ethanol for 24 hours prior to H&E staining. H&E staining was performed using a standard protocol of 2x3 minute washes in 100% ethanol, 2x3 minute washes in 96% ethanol, 2x3 minute washes in 70% ethanol, 3 minute wash in tap water, 3 minute staining in hematoxylin (Sigma-Aldrich, St. Louis, MO), 3 minute wash in distilled water, 30 second staining in eosin Y (Sigma-Aldrich), rinse with tap water until the water runs clear, 1 minute wash in xylene, and 30 second wash in ethanol. Slides were cover slipped and imaged at 40x magnification in brightfield on a Slideview VS200 Olympus Slide Scanner (Evident Scientific, Tokyo, Japan).

MS/MS of pure diGlu standard and the diGlu peak detected on kidney tissue sections of diGlu-treated mice was performed with nitrogen as collision gas, using 1000 shots across the m/z range of 50-1,000 Da. The laser power was set to 90% with an isolation window of ± 1 Da and a collision energy of 35 eV.

### Delayed renal histological assessment (48 h post-injection)

The experimental groups consisted of control mice, iopamidol-treated mice (1.5 g iodine kg^-1^), and diGlu-treated mice (750 mg kg^-1^). Kidney tissue was collected at 48 hours after injection for histological assessment.

Mouse kidneys were harvested immediately following euthanasia and briefly rinsed in cold phosphate-buffered saline (PBS) to remove residual blood. Tissues were immersion-fixed in 4% paraformaldehyde (PFA) at 4 °C for 48 hours to preserve morphology. Following fixation, kidneys were cryoprotected in 30% (w/v) sucrose prepared in 4% PFA at 4 °C until the tissue equilibrated and sank, indicating complete infiltration (~48 hours). Samples were then embedded in OCT compound, oriented to obtain longitudinal sections encompassing both cortex and medulla, and rapidly frozen on dry ice. Embedded tissues were stored at -80 °C until sectioning. Frozen kidney blocks were sectioned at 17 µm thickness using a cryostat maintained at approximately -20 °C, and sections were collected onto charged glass slides and allowed to air dry. For hematoxylin and eosin (H&E) staining, sections were rehydrated in PBS, stained with hematoxylin to visualize nuclei, rinsed and differentiated as needed, and counterstained with eosin to label cytoplasmic and extracellular components. Slides were then dehydrated through graded alcohols, cleared in xylene, and cover-slipped using DPX mounting medium prior to imaging.

### Statistical analysis

Data was analyzed using MATLAB and GraphPad Prism. RMSE was calculated between electrode pH and ratiometric CEST-derived pH. Group comparisons used unpaired two-sample t-tests with Welch’s correction (unequal variances). Data are reported as mean ± SD unless noted.

This study was designed as an exploratory feasibility assessment, and no formal a priori power calculation was performed. Sample sizes were selected based on our prior renal CEST imaging using diGlu, indicating that robust contrast would be achieved at the higher dose levels.

Because no formal a priori power calculation was performed, a post hoc power analysis was included as a supportive assessment. For the post hoc analysis, the main group comparisons were performed using the Wilcoxon rank-sum test (Mann-Whitney U test, non-parametric). A Welch’s t-test and permutation testing were also run as supporting checks, to see whether the findings held up across different approaches. Effect sizes were reported as Hedges’ g. Power analysis was performed for the primary comparisons using a two-sample t-test framework based on the observed effect size (Cohen’s d), sample size, and α = 0.05.

## Results

### Synthesis and physicochemical characterization

We needed to re-engineer the synthesis for higher yield and to remove potentially toxic and difficult to remove compounds such as TFA to perform large animal studies of diGlu which requires production of gram scale quantities. The streamlined three-step process described in **Figure 2** provides I45DC-diGlu in gram quantities with high purity and excellent batch-to-batch reproducibility. The key differences from prior phenyl-ester-based schemes^32^, ^33^ are (i) direct activation of imidazole-4,5-dicarboxylic acid to the 1H-imidazole-4,5-dicarbonyl dichloride under SOCl_2_ with catalytic DMF, (ii) low-temperature coupling (-80 °C → RT) of the di-acid chloride with L-glutamic acid di-tert-butyl ester·HCl (L-Glu(Ot-Bu)_2_·HCl) in dry THF under N_2_ to suppress side-product acyl migration, and (iii) acidic global deprotection using mild 12 M HCl in a biphasic DCM/H_2_O system, which obviates the need for TFA deprotection and avoids peptide backbone cleavage, thus preserving the imide/amide backbone. Conceptually, the logic of the reaction is to activate the diacid, then couple it with H-Glu(OtBu)_2_·HCl under a hindered base (DIPEA/EtNiPr_2_), followed by global deprotection. To accomplish this, we replaced the diphenyl ester intermediate and TFA workup with a direct acid-chloride route and HCl/DCM–water deprotection. The changes shorten cycle time, reduce by-products from phenyl esters, and maintain stereochemical integrity on glutamate side chains through low-temperature addition and excess DIPEA. Elimination of TFA did not impact the yield after deprotection. Low-temperature addition, excess hindered base (DIPEA), and anhydrous conditions suppressed acyl migration and oligomerization, yield a clean bis-amide after silica flash chromatography. Reaction monitoring by TLC and analytical HPLC indicated near-quantitative conversion at each step. After the final step, we produced I45DC-diGlu as a white powder with an 85% yield which was a significant improvement over the ~66% yield reported for phenyl-ester based I45DC syntheses.^33^ This workflow lowers reagent cost, shortens reaction time (3 vs. 5 days), and improves batch-to-batch reproducibility, facilitating gram-scale production for pre-clinical imaging.

### Biodistribution by MALDI mass spectrometry imaging

In preparation of MALDI imaging experiments, we performed MALDI target plate experiments to determine if the diGlu molecule ionizes with MALDI. This experiment was performed with DHB matrix in positive ion mode and showed that MALDI ionizes diGlu as protonated, sodiated and potassiated molecular ions at m/z of 415.1102 Da for [diGlu+H]^+^, m/z 437.0922 Da for [diGlu+Na]^+^ and m/z 453.0655 for [diGlu+K]^+^ as listed in **Table 1**.

**Figure 3:**
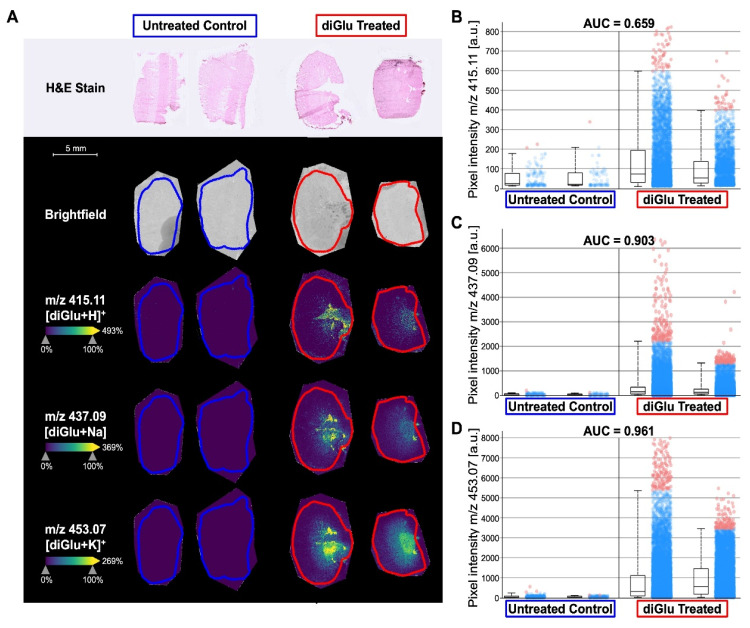
** MALDI-MSI showing renal biodistribution of I45DC-diGlu.** (A) H&E images (top) and corresponding ion maps (bottom) of kidneys from control (blue outline) and diGlu-treated mice (red outline) show intense [diGlu+H]⁺ = 415.11, [diGlu+Na]⁺ = 437.09 and [diGlu+K]⁺ = 453.07 signals confined to the papilla/medulla only after treatment. Scale bar = 5 mm. (B-D) Pixel-intensity box plots for each ion give ROC AUCs of 0.659, 0.903 and 0.961, confirming clear discrimination between treated and control tissue.

MALDI imaging of mouse kidney, liver, and pancreas from diGlu-treated *versus* control mice was measured on central coronal tissue sections. Kidney sections from diGlu-injected mice showed significant diGlu MALDI imaging signal at the expected m/z values of the ion adducts of protonated, sodiated and potassiated diGlu ions (**Figure 3**). Quantification from pixel intensity plots and box-and-whisker plots for each tissue section, and receiver operating characteristic (ROC) analyses per diGlu ion comparing pooled pixels per tissue and treatment groups demonstrated significantly increased diGlu levels in diGlu-treated *versus* control kidneys for [diGlu+Na]^+^ (AUC = 0.903) and [diGlu+K]^+^ (AUC=0.961), while [diGlu+H]^+^ was close to the significance level (AUC = 0.659). The overall signal intensity for [diGlu+H]^+^ was by a factor of about 10 lower in diGlu-treated mouse kidney sections than the signal intensities for [diGlu+Na]^+^ and [diGlu+K]^+^, which may explain the higher significance levels for the latter two ions. Since the MALDI imaging spectra of control kidney, pancreas, and liver showed no signal in any of the spectral regions of [diGlu+H]^+^ at 415.11, [diGlu+Na]^+^ at 437.09, and [diGlu+K]^+^ at 453.07, we concluded that there are no endogenous, overlapping tissue metabolites present as evident from **Figure 3** and Supplemental **Figures S2** and **S3**. Moreover, no additional MALDI imaging signals were detected in tissues from diGlu-treated animals other than the three detected molecular ion adducts from diGlu, indicating that no metabolized diGlu was present in any of the tested tissues.

As evident from the H&E stains of the same kidney tissue sections which had undergone MALDI imaging, the signal of all three diGlu ions was present in the central part of the kidney, indicating that the drug is passing through the medulla and not dispersing through the cortex (**Figure 3**). No diGlu MALDI imaging signal was observed in the pancreas and liver tissue sections of diGlu-treated mice, demonstrating that the drug passes through the kidney and is not retained in the liver or pancreas at 1-minute post-injection (**Figures S2, S3**).

**Figure 4:**
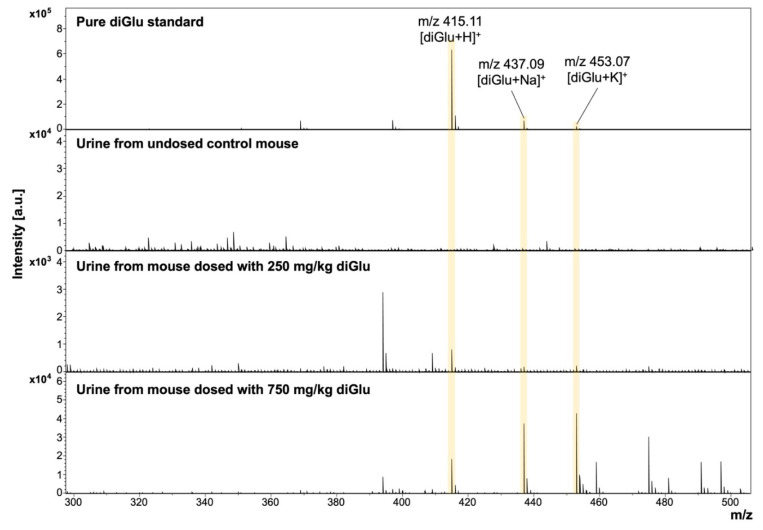
** MALDI target plate data of pure diGlu standard (top row) and mouse urine from control *versus* diGlu-treated mice (rows 2-4).** The diGlu standard shows the same signals as observed by MALDI imaging in Figure 3, which are [diGlu+H]^+^ at m/z 415.11, [diGlu+Na]^+^ at m/z 437.09, and [diGlu+K]^+^ at m/z 453.07. Urine from untreated control mice does not show any signals at these m/z values (second row). The urine from mice treated with diGlu at doses of 250 mg kg^-1^ (third row) and 750 mg kg^-1^ (bottom row) show the same MALDI signals as the diGlu standard, with the highest dose showing all three diGlu ions of protonated, sodiated, and potassiated diGlu (bottom row).

Urine analysis from diGlu-treated *versus* control mice showed that higher injected doses of diGlu resulted in higher urine levels of unmetabolized diGlu (**Figure 4**). This was evident from the detection of m/z of 415.11 Da for [diGlu+H]^+^ in the urine of mice intravenously injected with 250 mg kg^-1^ of diGlu (**Figure 4**). In the urine of mice intravenously injected with 750 mg kg^-1^ of diGlu, we detected all three diGlu ions at 415.1102 Da for [diGlu+H]^+^, m/z 437.0922 Da for [diGlu+Na]^+^, and m/z 453.0655 for [diGlu+K]^+^, which was confirmed by comparison with MALDI spectra of pure diGlu standard in the top row (**Figure 4**), as well as MALDI MS/MS fragmentation spectra shown in the supporting information (**Figures S4, S5**). [diGlu+Na]^+^ at 437.09 and [diGlu+K]^+^ at 453.07 were markedly elevated in the urine of animals dosed with 750 mg kg^-1^ of diGlu as compared to pure diGlu standard compound and urine from mice dosed with 250 mg kg^-1^ of diGlu, which likely resulted from higher concentrations of sodium and potassium in the urine of the 750 mg kg^-1^ diGlu-dosed mice as compared to pure diGlu standard and urine from 250 mg kg^-1^ diGlu-dosed mice. Pure diGlu standard did not contain significant amounts of sodium and potassium, therefore preferentially forming the protonated ion adduct. Urine from 250 mg kg^-1^ diGlu-dosed mice likely also contained significantly less sodium and potassium than urine from 750 mg kg^-1^ diGlu-dosed mice. This MALDI imaging data supports our contention that this imaging agent is predominantly eliminated by renal excretion and is excreted intact, which are the desired features for renal and tumor MRI pH imaging studies.

To extend the renal biosafety assessment beyond the immediate post-administration time point used for MALDI-associated histology, we examined kidneys collected at 48 hours after injection by H&E staining (**Figure S6**). Across control, iopamidol-treated (3,060 mg kg^-1^), and I45DC-diGlu-treated mice, renal architecture was preserved and no overt histopathological abnormalities were observed.

### *In vivo* dose-dependence of MRI contrast for diGlu

As seen in **Figure S7**, diGlu produces characteristic Z-spectra and MTR_asym_ spectra with two saturation dips at 7.7 ppm (imidazole NH) and 4.5 ppm (amide NH). Also similar to our previous studies,^33^ the ratio ST_4.5_/ST_7.7_ varied monotonically with pH and could be fit to a third-order polynomial. Power-dependence studies (B_1_= 2.0-8.0 µT) identified 5 µT as the optimum for maximal saturation transfer with minimal direct water saturation (**Figure S8**). Phantom validation against electrode pH yielded an RMSE of ~0.10 pH units under the same saturation settings (B_1_ = 5 µT, T_sat_ = 2 s). We expect this calibration based RMSE provides a solid estimate of the uncertainty in converting MTR_ratio_ to pH *in vivo* (in addition to measurement noise and residual field effects).

**Figure 5:**
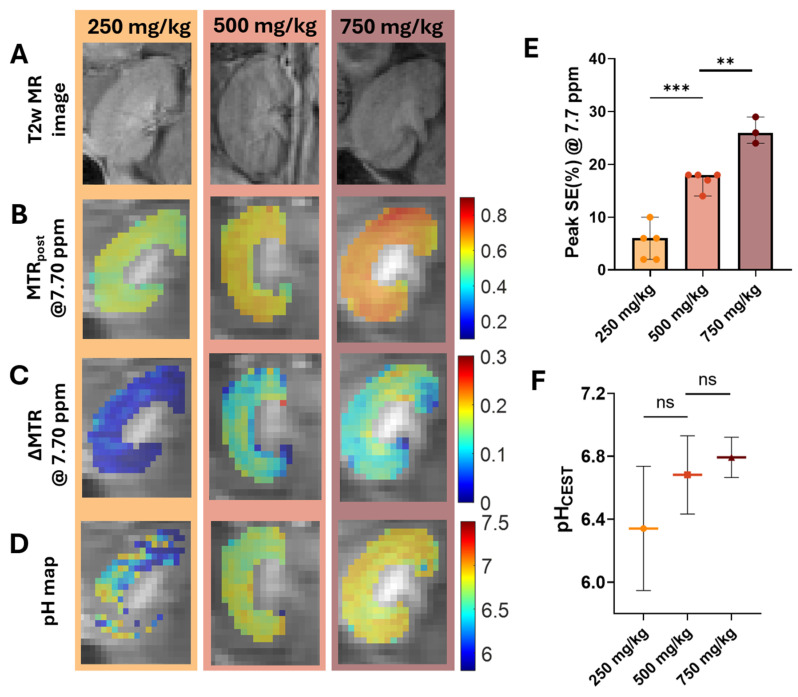
** Dose-dependent CEST MRI contrast in kidneys following administration of I45DC-diGlu.** Dose dependence of renal CEST contrast and pH readout with I45DC-diGlu. (A) Representative T_2_-weighted mouse kidney images; mice were injected intravenously with 250, 500, or 750 mg kg^-1^ I45DC-diGlu. (B) Post-injection MTR maps at 7.70 ppm (MTR) overlaid on M_0_. (C) ΔMTR at 7.70 ppm (post–pre) highlighting dose-dependent enhancement. (D) Ratiometric pH maps derived from ST_4.5_/ST_7.7_ after B_0_ correction (WASSR), showing greater visibility of corticomedullary acidification with increasing dose. (E) Peak signal enhancement at 7.7 ppm (SE%, relative to pre-injection baseline) increases from 250→500→750 mg kg^-1^ (mean ± SD, unpaired two-tailed t test with Welch’s correction; **p < 0.01, ***p < 0.001). (F) renal pH_CEST_ across doses (mean ± SD, unpaired two-tailed t test with Welch’s correction; ns-not significant). (Acquisition: 11.7 T, CW presaturation B₁ = 5 µT, t_sat_ = 2 s; single coronal kidney slice).

We next moved to injecting multiple doses of diGlu into healthy mice and collecting MRI for evaluating the dose dependence of the contrast. C57BL/6J mice (24-26 g) received tail-vein injections of diGlu at 250 mg kg^-1^ (n = 5), 500 mg kg^-1^ (n = 5), or 750 mg kg^-1^ (n = 3). Because the ratiometric readout uses signals at both 4.5 and 7.7 ppm, we injected these doses to ensure adequate SNR at both offsets to attain stable ST_4.5_/ST_7.7_ (MTR_ratio_) maps. The *in vivo* CEST MRI workflow is shown in **Figure S9A** using the sequence shown in **Figure S9B** and enabled ROI-wise kinetic analysis and pH mapping. Representative kidney T_2_-weighted images and corresponding parametric maps are shown in **Figure 5A-D.** Post-injection MTR_post_ at 7.7 ppm and the corresponding ΔMTR increased with dose (**Figure 5B,C**) and peak ΔMTR at 7.7 ppm rose significantly across groups (**Figure 5E**; Welch’s t-test, 250 vs 500 mg kg^-1^, p < 0.001, 500 vs 750 mg kg^-1^, p < 0.01). Ratiometric pH maps (**Figure 5D**) displayed improved resolution at higher doses and revealed the expected corticomedullary gradient; group summaries (**Figure 5F**) indicated similar pH measurements between doses. Across animals, the outer medulla (OM) displayed the highest peak SE, followed by cortex and inner medulla (IM) consistent with corticomedullary flow and trapping differences. These findings suggest that diGlu exhibits high CEST sensitivity and dose-responsive contrast behavior, with robust detectability at 500 mg kg^-1^ and above for both exchangeable proton pools.

### Regional kidney pH mapping

**Figure 6:**
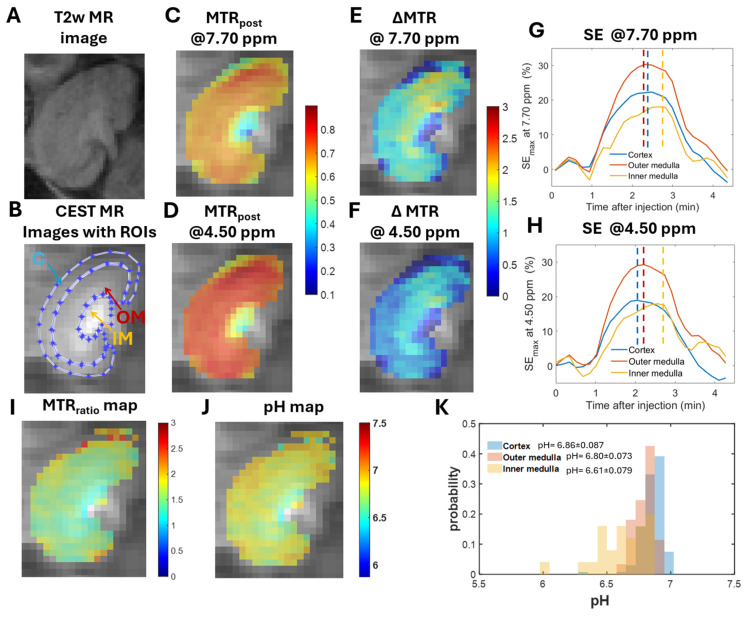
** Regional kidney pH mapping using I45DC-diGlu (750 mg/kg) reveals physiological pH gradients across renal compartments.** Regional CEST contrast dynamics and ratiometric pH mapping in mouse kidney after I45DC-diGlu. (A) T2-weighted anatomy. (B) CEST image with ROIs for cortex (C, blue), outer medulla (OM, red), and inner medulla (IM, yellow). (C, D) Post-injection MTR maps at 7.70 ppm and 4.50 ppm (MTR_post_). (E, F) ΔMTR maps (post–pre) at 7.70 and 4.50 ppm showing stronger enhancement in outer medulla > cortex > inner medulla. (G, H) Signal-enhancement (SE%) time courses for each ROI at 7.70 and 4.50 ppm; dashed lines mark time-to-peak for cortex (blue), outer medulla (red), and inner medulla (yellow), illustrating faster cortical wash-in and slower medullary kinetics. (I) MTR_ratio_ map (7.70/4.50) used for pH estimation (J) Pixel-wise pH map derived from the ST_4.5_/ST_7.7_ ratio, revealing the expected corticomedullary acidification gradient. (K) ROI histograms with mean ± SD: cortex 6.86 ± 0.087, outer medulla 6.80 ± 0.073, inner medulla 6.61 ± 0.079.

We then moved to investigating the distribution of pH values within the kidneys based on our diGlu MRI data in healthy mice.** Figure 6** summarizes the derivation of regional pH and the resulting spatial pattern. ROI masks for cortex (C), outer medulla (OM), and inner medulla (IM) were drawn on the CEST reference image (**Figure 6B**) and applied to all parametric maps. The MTR_post_ and ΔMTR maps at 7.70 ppm and 4.50 ppm (**Figure 6C-F**) demonstrate robust post-injection contrast at both offsets, highlighting the renal medulla while preserving cortical signal. From these maps, we computed the MTR_ratio_ map (**Figure 6I**) and converted it voxelwise to pH using the phantom-derived ratiometric calibration (**Figure 6J**) under the same B_1_ = 5 µT and T_sat_ = 2s used *in vitro* and |ΔB_0_| ≤ 0.3 ppm. The voxel distributions show a clear cortico-medullary gradient (C > OM > IM). Histogram summaries (**Figure 6K**) quantify this ordering (mean ± SD): cortex- 6.86 ± 0.087, outer medulla- 6.80 ± 0.073, inner medulla- 6.61 ± 0.079 (mean ± SD). Time-course plots at 7.70 ppm and 4.50 ppm (**Figure 6G,H**) show consistent uptake and washout across ROIs; peak times (dashed lines) follow the same gradient (cortex fastest, inner medulla slowest), providing an internal dynamic check that supports the static pH maps. To compare how this agent performs with iopamidol, we collected CEST MRI pH maps with the results shown in supplemental **Figure S10**. As can be seen, peak saturation enhancement was similar when injecting ~4 xs the dose of iopamidol (3,060 mg kg^-1^) compared to diGlu (750 mg kg^-1^) with renal DMTR_asym_ ~25% for the whole kidneys. pH values of cortex- 6.64 ± 0.085, outer medulla- 6.64 ± 0.049, inner medulla- 6.61 ± 0.081 (mean ± SD) obtained for iopamidol. Across animals, OM exhibited the highest peak SE with intermediate pH, consistent with known corticomedullary flow and tubular handling. Altogether, the maps and distributions establish a physiologically plausible pH gradient (C > OM > IM) with good spatial coverage and internal consistency across the two offsets. We did not perform invasive *in vivo* pH validation (e.g., microelectrode) in the same animals.

### ROI-wise PK data, modeling and model selection

**Figure 7:**
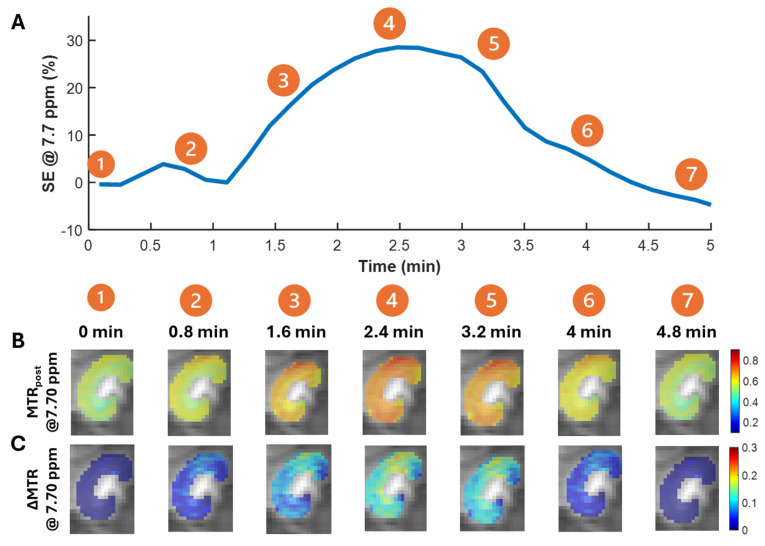
** Dynamic CEST MRI reveals rapid renal clearance kinetics of I45DC-diGlu.** (A) Whole-kidney signal-enhancement (SE%) at 7.7 ppm versus time (pre-injection baseline = 0%); numbered markers correspond to the image panels below. (B) Representative post-injection MTR_post_ maps at 7.7 ppm at seven time points (0, 0.8, 1.6, 2.4, 3.2, 4.0, and 4.8 min), overlaid on M0 images. (C) Corresponding ΔMTR maps (post–pre) at 7.7 ppm showing rapid wash-in to peak contrast (~2.4–3.2 min) followed by wash-out by ~5 min.

Finally, we moved to characterizing the renal uptake and excretion of diGlu through MRI signal changes.** Figure 7** summarizes the dynamic changes in CEST MRI contrast at 7.70 ppm for mice injected with 750 mg kg^-1^ of diGlu. The global SE(%) curve (**Figure 7A**) shows a rapid uptake within the first minute, a short plateau with peak at ~2.4-3.2 min, and a mono-exponential-like decline toward baseline by ~4.2 min. Matching snapshots of MTR_post_ and ΔMTR at seven marked times (**Figure 7B,C**) visualize this course: enhancement appears first along the cortical rim (0-1.6 min), spreads into the outer medulla around the peak (≈2.4-3.2 min), and recedes during wash-out. Regionally, contrast emerges earliest and strongest in cortex and outer medulla, while the inner medulla remains comparatively lower and begins to fade first during clearance. By the final time point, most of the kidney parenchyma approaches pre-injection signal levels as expected. This provides a qualitative, voxel-wise view of the spatiotemporal enhancement pattern in a representative 750 mg kg^-1^ experiment.

**Figure 8:**
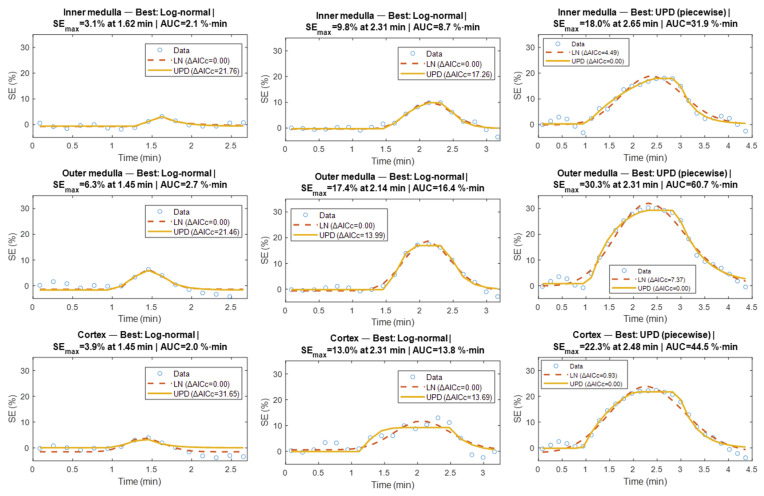
** ROI-based pharmacokinetic modeling of dynamic renal CEST MRI data across dose, modeled with Log-normal vs. Uptake–Plateau–Decay (UPD).** Signal-enhancement (SE, %) time courses from inner medulla, outer medulla, and cortex are shown for 250, 500, and 750 mg kg^-1^. Open circles denote measured data. Dashed curves are log-normal (LN) fits; solid curves are UPD (piecewise) fits. The best model in each panel was selected by corrected Akaike information criterion (ΔAICc = 0 indicates the preferred model). Increasing dose yields larger SEₘₐₓ and AUC and broader kinetics, with a shift from LN-like bolus curves at 250–500 mg kg^-1^ to UPD-dominated behavior at 750 mg kg^-1^, particularly in outer medulla and cortex, indicating stronger uptake/retention at higher dose. Abbreviations: IM, inner medulla; OM, outer medulla; LN, log-normal; UPD, Uptake–Plateau–Decay; SEₘₐₓ, signal enhancement with time-to-peak, min; AUC, area under curve (baseline-referenced, %·min); ΔAICc, difference from the best AICc.

To extract quantitative parameters from the data for quantifying diGlu PK, two candidate models were evaluated and compared - the LN model and UPD model. We chose these models based on the shape of the contrast curves, as the LN model captures unimodal bolus-like uptake/clearance, whereas the UPD model captures rise-plateau-washout behavior seen in some ROIs. **Figure 8** summarizes representative ROI fits across doses, and **Table S1** compiles the corresponding parameters and goodness-of-fit (GOF) metrics, enabling quantitative comparison of renal kinetics between regions and dose levels. For a given dosage, the preferred model was chosen by AICc (ΔAICc shown in legends). At 250 mg kg^-1^, enhancement was small (SE_max_ ≈ 3-6%) with shortest TTP (TTP ≈ 1.45-1.62 min), and the LN model consistently outperforming UPD for each mouse (ΔAICc ≫ 2), reflecting a unimodal rise-decay with no discernible plateau phase (**Figure 8**, left column; Table S1). At 500 mg kg^-1^, the signal was much stronger (SE_max_ ≈ 10-18%; TTP ≈ 2.14-2.31 min) and LN remained the best model for all mice, although UPD provided competitive fits (ΔAICc ≈ 14-32 vs LN). At 750 mg kg^-1^, the curves displayed a clear plateau with prolonged washout (SE_max_ ≈ 18-30%; TTP ≈ 2.31-2.65 min), and UPD became the preferred model in all ROIs (ΔAICc vs LN ≈ 3.9-13.1), capturing the rise-hold-decay behavior (**Figure 8**, right column). Consistent with the spatial analyses, outer medulla (OM) exhibited the largest exposure and fastest buildup at higher doses, followed by cortex (C) and inner medulla (IM). Furthermore, we observed:

- SE_max_. Median values rose monotonically with dose (Table S1): 250 mg kg^-1^: ~3-6% → 500 mg kg^-1^: ~10-18% → 750 mg kg^-1^: ~22-30% (OM highest).
- TTP. Increased modestly with dose, from ~1.45-1.62 min (250 mg kg^-1^) to ~2.31-2.65 min (750 mg kg^-1^), consistent with higher delivered mass and transient plateau formation.
- Uptake Slope (%·min^-1^). Steepened with dose (e.g., cortex examples ~0.95-4.28 → 8.3-6.9 → 12.7-16.5) indicating faster early delivery at higher dosing.
- t_1/2_ (min). Increased with dose (≈ 1.6-1.9 min at 250 mg kg^-1^ → 2.5-2.8 min at 500 mg kg^-1^ → 3.2-3.3 min at 750 mg kg^-1^), in line with the prolonged decay.
- AUC_total_ (%·min). Increased strongly with dose and region: ~2-3 (250 mg kg^-1^) → ~14-32 (500 mg kg^-1^) → ~45-61 (750 mg kg^-1^), with OM > C > IM at 750 mg kg^-1^.

**Figure S11** also provides group-level summaries of regional pH and pharmacokinetic parameters across dose levels. Overall, the CEST-derived pH measurements stayed fairly stable across doses and continued to show the expected corticomedullary pattern with lowest pH observed in the inner medulla. The SE_max_, TTP, t_1/2_, and AUC all increased in a statistically significant manner with increasing dose. Across the renal compartments, the outer medulla consistently exhibited the strongest enhancement and the highest cumulative exposure. Post hoc analysis demonstrated dose-related differences in AUC between adjacent groups (Table S2). AUC was significantly higher in the 750 mg kg^-1^ group than in the 500 mg kg^-1^ group (mean difference: 9.33, 95% CI: 4.25–14.42; median difference: 8), with significance observed in the primary Wilcoxon rank-sum test (*p* = 0.0357) and supported by permutation testing (*p* = 0.0186) and Welch’s *t*-test (*p* = 0.00935). Similarly, the 500 mg kg^-1^ group exhibited significantly higher AUC than the 250 mg kg^-1^ group (mean difference: 12.60, 95% CI: 8.21–16.99; median difference: 16), with consistent significance across the Wilcoxon rank-sum test (*p* = 0.00794), permutation testing (*p* = 0.0082), and Welch’s *t*-test (*p* = 0.000468). Importantly, both comparisons showed large effect sizes, with Hedges’ *g* values of 4.00 and 4.05, respectively. Post hoc power was calculated using the observed effect size (Cohen’s d), sample size, and α = 0.05 within a two-sample t-test framework. Observed power was high for both comparisons (>0.99), reflecting the large effect sizes observed between dose groups.

Goodness-of-fit (AICc, R², RMSE) and residual analyses support the selected LN/UPD models across doses and renal regions. Goodness-of-fit values were high for both models when appropriate: at 500-750 mg kg^-1^, R²_UPD_ frequently reached 0.96-0.99 with RMSE_UPD_ ≈ 1.0-1.5 SE%; at 250 mg kg^-1^, R²_LN_ ≈ 0.62-0.93 with RMSE_LN_ ≈ 0.7-1.9 SE%. The AICc criterion clearly separated regimes: LN for low dose (featureless unimodal curves), UPD for highest dose (visible plateau). Residual diagnostics (**Figure S12**) showed near-zero-centered SE% residuals without obvious temporal structure across cortex, outer medulla, and inner medulla for both LN and UPD fits, supporting model adequacy. Residual histograms (**Figure S13**) were approximately Gaussian across doses, with narrower dispersion for UPD in medullary ROIs, consistent with its lower AICc in those regions. Together, these results support a strong increase in AUC across the tested dose range. The emergence of an UPD-like shape at high dose likely reflects transit and transient retention within medullary/tubular compartments prior to clearance, whereas the LN-like behavior at low dose is consistent with fast, dilute bolus passage. The ROI-wise kinetics support a picture of rapid renal delivery, short retention and rapid kidney clearance of intact diGlu for all doses, with a dose-dependent plateau formation at 750 mg kg^-1^ and largest exposure in the OM. To further evaluate the robustness of the UPD kinetic model, residual diagnostics were performed across renal compartments for dosage of 750 mg kg^-1^(Figure S14). Residual frequency distributions were centered around zero, without marked skew, suggesting no strong systematic bias in model predictions. Autocorrelation analysis showed moderate but non-uniform lag-1 autocorrelation (ACF_1_ ≈ 0.26-0.58) across the cortex, outer medulla, and inner medulla, while higher-lag autocorrelations were generally within the confidence bounds. These findings indicate that the local deviations in the fitted curves, including the fluctuation near the plateau-to-washout transition, likely reflect transient variability rather than systematic model instability, supporting the robustness and physiological plausibility of the UPD model.

To evaluate the translational potential of diGlu, we performed a set of 3 T and 11.7 T Bloch simulations using the exchange rates measured previously.^9^ The results are shown in **Figure S15**. These simulations displayed lower contrast at 3 T using a lower-power saturation (2.4 μT, 3 s) compared to what would be expected at 11.7 T using the saturation applied in this study (11.7 T, 5 μT, 2 s), however the dual-offset ratiometric readout remained monotonic across the physiologic pH range and enabled accurate polynomial fitting to calibrate the ST_ratio_ dependence on pH. The fitted RMSE was 0.028 pH units at 11.7 T and 0.045 pH units at 3 T, indicating that quantitative ratiometric pH mapping remains feasible under clinically relevant lower-power conditions, despite a reduced dynamic range.

## Discussion

We report a practical, high-yield route to produce diGlu together with the first ROI-wise pharmacokinetic (PK) modeling framework for renal CEST MRI dynamics which displays rapid intact clearance of this probe with the clearance rate impacted by the dosage. The re-engineered synthesis achieved a high yield for a promising MRI pH imaging agent (diGlu). Our three-step synthesis utilized only reagents that are ICH Q3A/B compliant with no heavy metals or controlled substances, phenyl-ester intermediates and TFA deprotection, all while boosting the yield from ~66% 32, 33 to 85%, which should streamline for CMC filings. We prepared gram-scale batches (≥ 3 g) reproducibly with overall impurity levels < 0.5% by HPLC. Collectively, the benign excipient profile and favorable physicochemical properties position diGlu as an attractive scaffold for translation.

We also report MALDI imaging data to demonstrate the biodistribution of this probe within the kidneys, pancreas and liver, which is a novel method and allows imaging of the distribution of the diGlu probe within the organ. MALDI mass-spectrometry imaging is increasingly used in PK/PD studies to map parent drug and metabolites directly in tissue, resolving regional heterogeneity that bulk LC-MS cannot capture.^43^ While absolute quantification remains sensitive to ion suppression and matrix effects, on-tissue calibration and stable-isotope internal standards are common strategies to improve accuracty.^44^, ^45^ Our MALDI data corroborated the predominant localization of diGlu within the kidneys with minimal uptake in the liver and pancreas, underscoring glomerular filtration as the dominant clearance pathway. No acute physiological perturbations were observed from our H&E data. The spatial localization of diGlu, which was detected in the central part of the kidney, likely the inner medulla, strongly indicates a non-toxic distribution of diGlu based on a recent publication by Chen *et al.*, which correlated the distinct renal spatial distribution of toxic and non-toxic compounds with *in vivo* nephrotoxicity.^46^ Localization of systemically administered compounds to the inner medulla of kidneys correlated with low or no nephrotoxicity in this study which tested a large variety of compounds.

Functionally, diGlu has two well-separated CEST offsets (7.7 and 4.5 ppm) that have suitable exchange rates and can be employed for robust ratiometric pH mapping with minimal spectral overlap. The ratiometric calibration of I45DC-diGlu enabled accurate pH estimation across the physiologic range, with low fitting error supporting its use for quantitative renal pH mapping. Higher doses (≥ 500 mg kg^-1^) improved spatial definition and revealed the expected cortico-medullary gradient (cortex > outer medulla > inner medulla). We furthermore clarified that doses of 750 mg kg^-1^ were well tolerated by mice, and that at this dose there is a slight plateau and prolonged retention. 750 mg kg^-1^ I45DC-diGlu showed similar renal contrast performance in healthy mice to ~3,060 mg kg^-1^ iopamidol which is the dosage used in a number of prior iodinated diaCEST studies.^9^ The pH values we measured in various regions of the kidney compare favorably with the pH values produced by Sun, Longo, Aime and colleagues in previous studies. 24 Renal MRI pH mapping utilizing triiodobenzenes has been utilized extensively in preclinical studies for detecting changes in kidney function including in ischemia reperfusion induced injury models, methyl malonic acid disease model, unilateral ureteral obstruction (UUO) models.^10^ The non-invasive and precise resolution of compartmentalized gradients positions diGlu as the contrast agent of choice for early diagnosis, functional assessment, and longitudinal therapy monitoring in these disorders. Ongoing work will evaluate the probe in models of metabolic acidosis, ischemia-reperfusion, MMA, and fibrosis, and refine sequence timing to exploit the 158 s half-life for dynamic renal-function assays. All of this further supports the potential of I45DC contrast agents as an alternative for renal pH mapping.

Another contribution of our work is methodological. Prior renal CEST reports emphasized static pH maps; here we propose and implement a data-driven PK modeling framework tailored to CEST signal-enhancement (SE%) time-courses. We fit each ROI with two complementary models: a LN and a piecewise UPD model and selected the preferred description by AICc on the SE time-course curves. This methodology yielded interpretable metrics (SE_max_, TTP, AUC_total_, t_1/2_) which could also help clarify kidney function along with pH. Empirically, the LN model best captured low-dose curves with unimodal kinetics, whereas UPD dominated at 750 mg kg^-1^, where a plateau and prolonged washout were evident. Regionally, outer medulla showed the largest exposure (AUC) and fastest buildup at higher doses, while inner medulla exhibited shorter half-times and smaller AUC, consistent with rapid tubular transit toward the papilla. To our knowledge, this is the first PK modeling of renal dynamics using CEST MRI, providing a reusable template for dose finding, protocol optimization, and future mechanistic (arterial input function, AIF-based) modeling.

There are several limitations to our work. One limitation is that we performed our experiments in male mice at 11.7 T, whereas translation of diGlu to humans at 3 T where Δω is smaller and direct water saturation is higher would require some advancements in signal acquisition and a full toxicology evaluation. Simulations further indicated that absolute CEST contrast is reduced under lower-power 3 T conditions, but the dual-offset ST_ratio_ remains monotonic across the physiologic pH range (**Figure S15**). This supports the feasibility of clinical translation with SAR-conscious optimization of saturation power and duration. This study reports baseline renal pH mapping in healthy mice; disease models and physiologic challenges (e.g., NaHCO_3_ or NH_4_Cl) were not included and will be addressed in future work. Establishing this baseline ratiometric performance is intended to enable subsequent studies of pH dysregulation in renal disease and treatment response. In this study, no acute adverse events were observed during imaging, and H&E at the sampled timepoints did not show any overt histopathological abnormalities. However, this study did not measure serum creatinine or blood urea nitrogen, both of which are important biomarkers for renal safety assessment. Other limitations of this study are the use of single-slice for dynamics, modest cohort sizes, and the absence of measuring arterial input function (AIF). With that said, the diGlu probe’s wide dual-offset window, rapid clearance, and iodine-free composition support applications ranging from early CKD acid-base assessment to radiation-free dynamic urography and peri-transplant graft monitoring. The benign excipient profile and absence of iodine or metals are favorable for translation; however, formal GLP tox, PK/PD, and 3 T protocol optimization are prerequisites for clinical evaluation. We are also testing (i) adiabatic frequency-selective pulses and (ii) partial deuteration to slow k_ex_ (~1,000 s^-1^); both to improve phantom signal-to-noise ratio (SNR) at 3 T. Structural analogs bearing alternative amino acids or isotope labels (¹³C, D) are also under evaluation to further tune exchange rates and boost clinical-field SNR. We will also evaluate inclusion of AIF compartment modeling to decouple delivery (*K^-1^*) and efflux (*k_2_*), multi-slice coverage with motion correction, powered cohorts for dose-response statistics, and 3 T optimization (frequency-selective saturation and timing tuned to the observed t_1/2_). Because of rapid kidney uptake (within 2.5 min to peak contrast), we don’t expect B_0_ drift to be an issue. However, a more complete Z-spectral collection with more shifts could be applied in cases with slower tissue contrast uptake and would allow time-resolved B_0_ monitoring/correction during the dynamic scan as performed in other studies.^9^ For larger animals and clinical translation, dosing should be considered on a molar basis and balanced against SAR and scan-time constraints, motivating protocol optimization to maintain ratiometric precision at lower administered doses. We also need to validate our PK modeling on perturbation models (metabolic acidosis, ischemia-reperfusion, UUO, methylmalonic acidemia, fibrosis). Nevertheless, we believe the diGlu platform is a promising one for kidney and cancer imaging.

## Conclusion

In this work we developed a robust synthesis of a promising pH imaging probe (diGlu) and established the biodistribution and PK of this probe using MALDI imaging and PK modeling of CEST MRI data. We also demonstrate that we can use this probe to display a corticomedullary gradient (cortex > outer medulla > inner medulla) in the pH maps. To our knowledge, this is the first ROI-wise PK modeling framework developed to quantify renal uptake and clearance using CEST MRI. The rapid clearance displayed by our renal PK modeling establishes diGlu as a practical and robust contrast agent for quantitative renal and cancer pH mapping.

## Supplementary Material

Supplementary methods, figures and tables.

## Figures and Tables

**Table 1:** Molecular diGlu ions observed by MALDI.

Ion	Observed m/z	Predicted m/z
[diGlu+H]^+^	415.11	415.11
[diGlu+Na]^+^	437.09	437.09
[diGlu+K]^+^	453.07	453.07

## Abbreviations

AICc: corrected Akaike information criterion
ΔAICc: AICc_LN_ - AICc_UPD_
AUC: area under the time-intensity curve
B_0_: main magnetic field
B_1_: RF saturation field amplitude
C: renal cortex
CEST: chemical exchange saturation transfer
CI: confidence interval
DCM: dichloromethane
DIPEA: N,N-diisopropylethylamine
DMF: N,N-dimethylformamide
DMSO-d_6_: deuterated dimethyl sulfoxide
ΔMTR: post-pre change in MTR
Δω: saturation frequency offset (ppm)
FOV: field of view
HPLC: high-performance liquid chromatography
I45DC-diGlu (diGlu): imidazole-4,5-dicarboxamide diglutamate
UUO: Unilateral Ureteral Obstruction
IM: inner medulla
k_ex_: chemical exchange rate
LN: log-normal (three-parameter) model
MALDI: matrix-assisted laser desorption/ionization
MRI: magnetic resonance imaging
MTR: magnetization transfer ratio
MTR_asym_: asymmetric magnetization transfer ratio
NEX: number of excitations
NMR: nuclear magnetic resonance
OM: outer medulla
pH_CEST_: pH estimated from ratiometric CEST
ppm: parts per million
ROI: region of interest
SE (%): signal enhancement at a given offset
SE_max_: peak signal enhancement
t_1/2_: time to half-decay since time of injection
T_sat_: saturation duration
TE: echo time
TR: repetition time
UPD: uptake-plateau-decay model
WASSR: water saturation shift referencing
Z-spectrum (Z): water signal vs Δω under saturation
H&E: Hematoxylin and Eosin
SNR: signal-to-noise ratio
