# Supplementary material for: Quantitative MRI Pharmacokinetic Modeling and MALDI Mass Spectrometry Imaging Biodistribution of a pH Mapping MRI Contrast Agent
Source: Theranostics. 2026 Jul 5;16(14):7937–57. doi: 10.7150/thno.128499 (PMC13440279; doi:10.7150/thno.128499)
Supplement: Supplementary file 1 [file thnov16p7937s1.pdf]

Supplementary information for

Quantitative MRI Pharmacokinetic Modeling and MALDI Mass Spectrometry Imaging Biodistribution of a pH Mapping MRI Contrast Agent

Synthesis Protocol

The synthesis and purification of I45DC-diGlu involved a six-step process, starting from imidazole-4,5-dicarboxylic acid (I45DC) and yielding the final diglutamate derivative with high purity suitable for in vivo studies after HPLC purification.

HPLC Purification

Final purification of the deprotected I45DC-diGlu was achieved via preparative high-performance liquid chromatography (HPLC). Approximately 50 mg of compound was dissolved in a 1:1 acetonitrile: water solution (50 mL) and 5 mL was loaded per run. The chromatographic separation was performed on a C18 column (5 µm) with a 29.5-minute gradient elution (flow rate 5 mL/min, 2 Hz sampling, ~2400 psi). The gradient ranged from 90:10 water: acetonitrile to 10:90, then returned to initial conditions. Collected fractions were lyophilized, yielding highly pure I45DC-diGlu.

Figure S1A: 1H NMR spectra

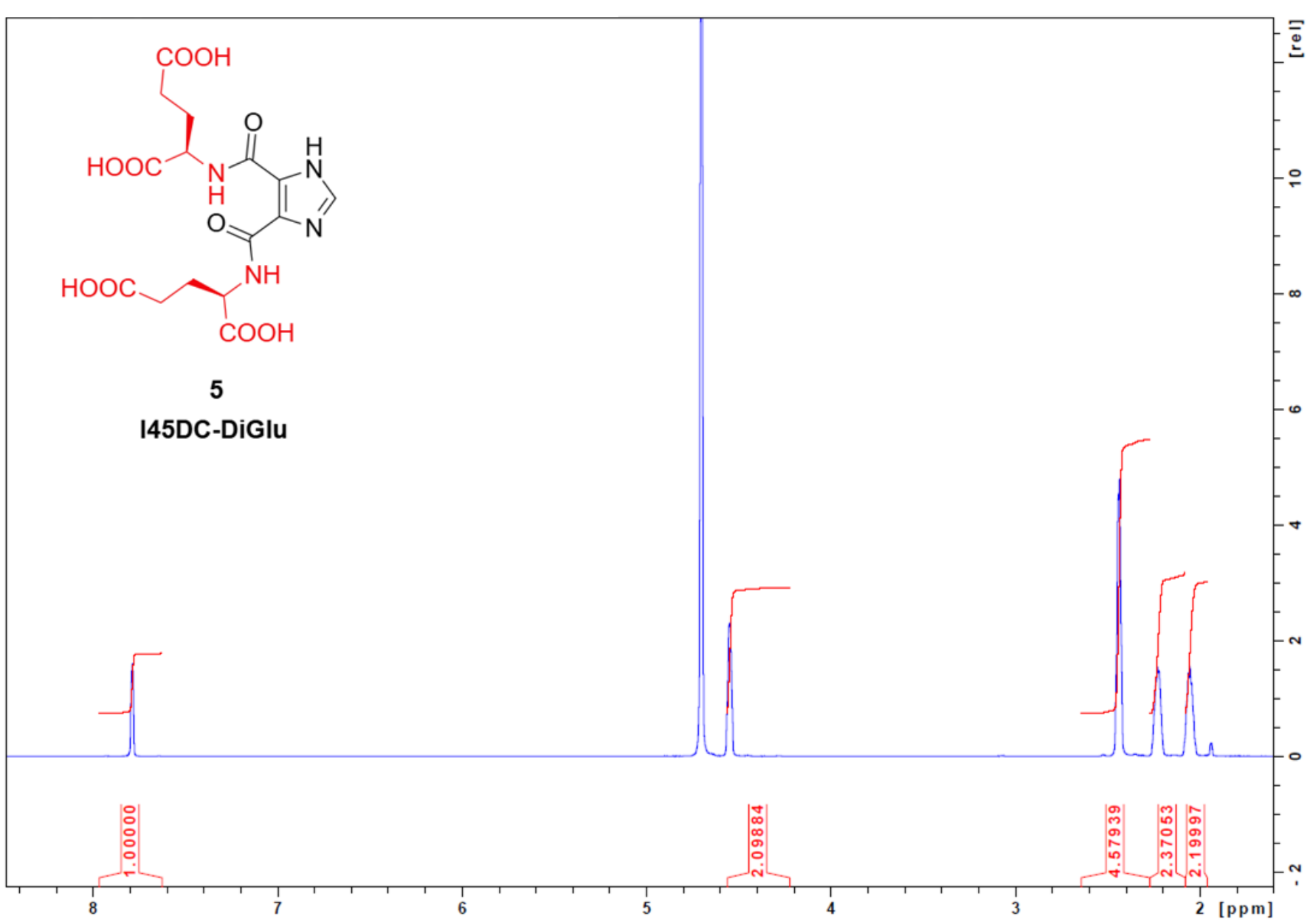

Figure S1B: HPLC chromatogram of the final injected I45DC-diGlu compound.

HPLC was performed using MP-A (0.1% formic acid in H_2O) and MP-B (0.1% formic acid in ACN) at a flow rate of 0.7 mL/min. The gradient was 95% A/5% B from 0 to 0.5 min, ramped to 59% A/41% B at 4.5 min, to 5% A/95% B at 5.5-6 min, and returned to 95% A/5% B at 6.5-8 min.

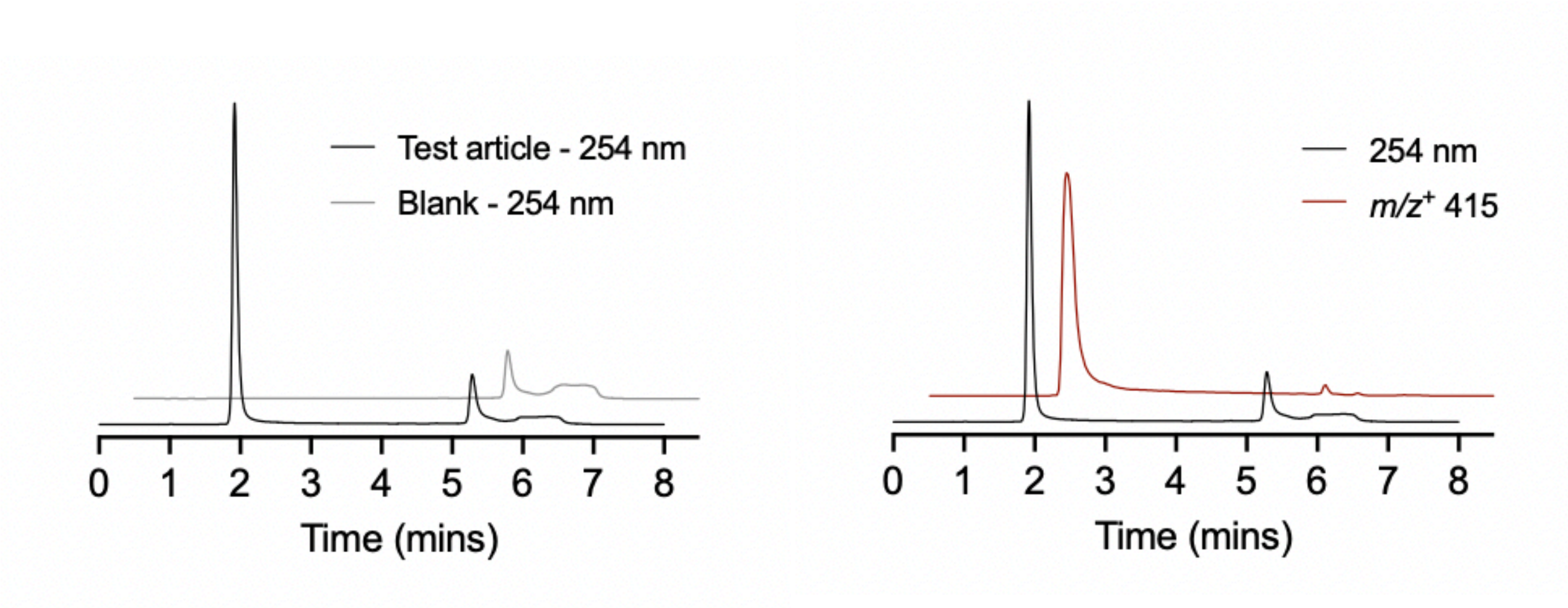

Figure S2: MALDI imaging of livers from control *versus* diGlu-treated mice.
(**A**) H&E stains (top row), brightfield images (second row), and MALDI images of the three ions generated from diGlu which are [diGlu+H]$^+$ at m/z 415.11 (third row), [diGlu+Na]$^+$ at m/z 437.09 (fourth row), and [diGlu+K]$^+$ at m/z 453.07 (bottom row) of central liver sections from two untreated control mice (blue outlines) *versus* two diGlu treated mice (red outlines). Quantification of MALDI images as box-and-whisker plots and the corresponding pixel intensity plots comparing liver images shown in (A) for control *versus* diGlu treated mice for (**B**) [diGlu+H]$^+$ at m/z 415.11, (**C**) [diGlu+Na]$^+$ at m/z 437.09, and (**D**) [diGlu+K]$^+$ at m/z 453.07. Blue dots are within the four quartile ranges and red pixels are outliers. AUC values from ROC curve analyses are reported as well.

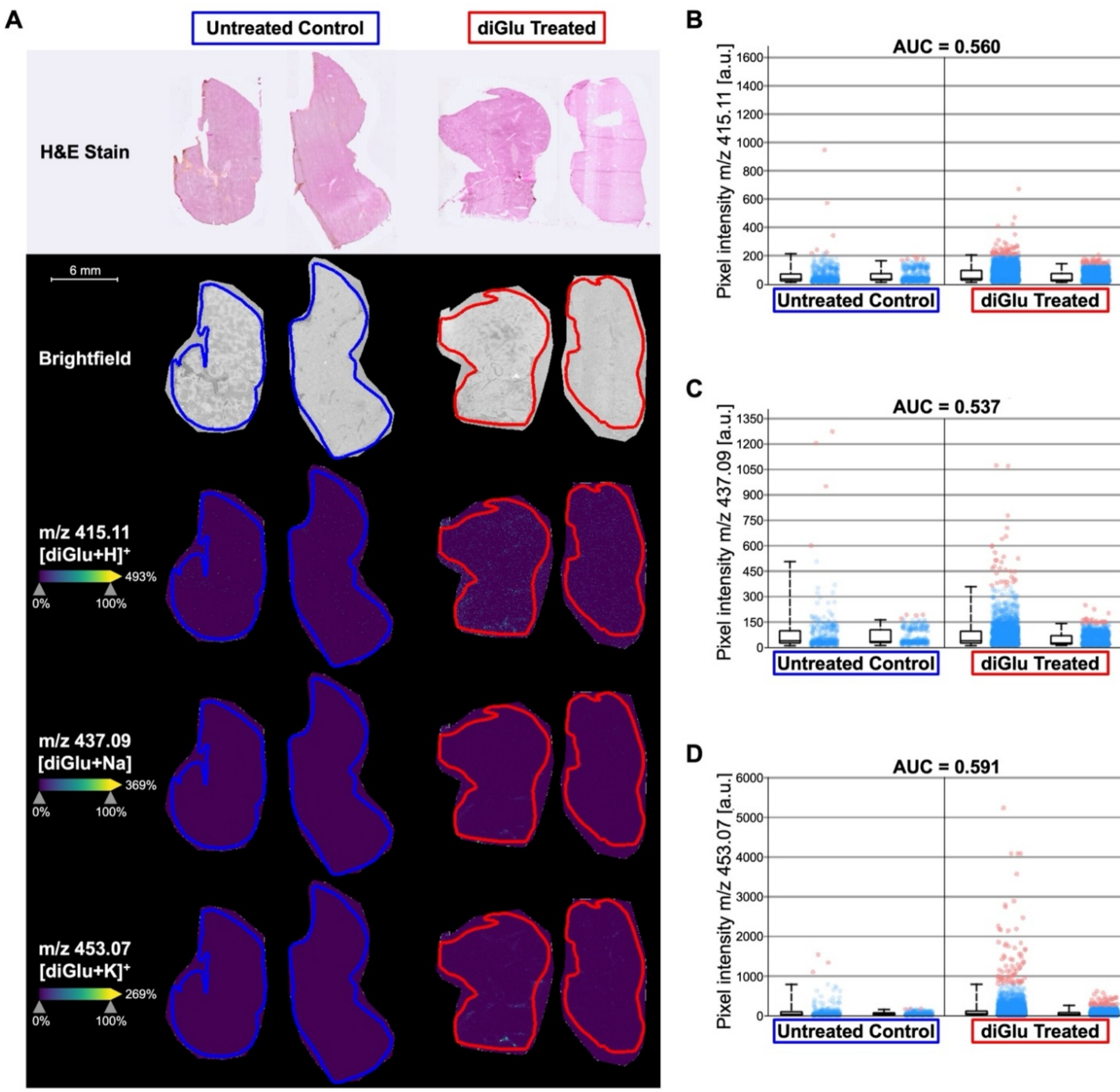

Figure S3: MALDI imaging of pancreases from control *versus* diGlu-treated mice.

(**A**) H&E stains (top row), brightfield images (second row), and MALDI images of the three ions generated from diGlu which are $[diGlu+H]^+$ at m/z 415.11 (third row), $[diGlu+Na]^+$ at m/z 437.09 (fourth row), and $[diGlu+K]^+$ at m/z 453.07 (bottom row) of central pancreas sections from two untreated control mice (blue outlines) *versus* two diGlu treated mice (red outlines). Quantification of MALDI images as box-and-whisker plots and the corresponding pixel intensity plots comparing pancreas images shown in (A) for control *versus* diGlu treated mice for (**B**) $[diGlu+H]^+$ at m/z 415.11, (**C**) $[diGlu+Na]^+$ at m/z 437.09, and (**D**) $[diGlu+K]^+$ at m/z 453.07. Blue dots are within the four quartile ranges and red pixels are outliers. AUC values from ROC curve analyses are reported as well.

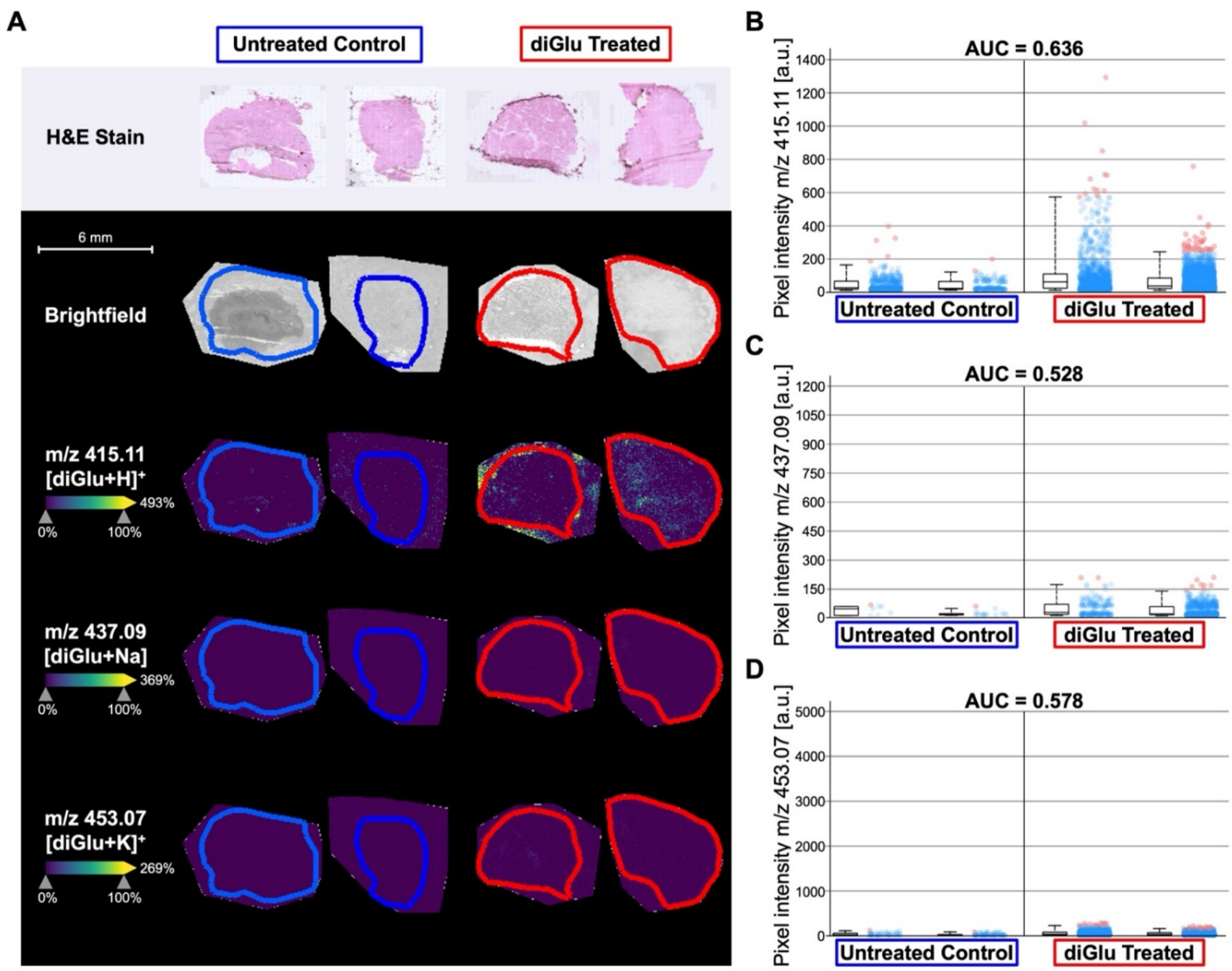

Figure S4: MS/MS fragmentation spectra of $[diGlu+H]^+$ from pure diGlu standard (top) and from kidney tissue (bottom).

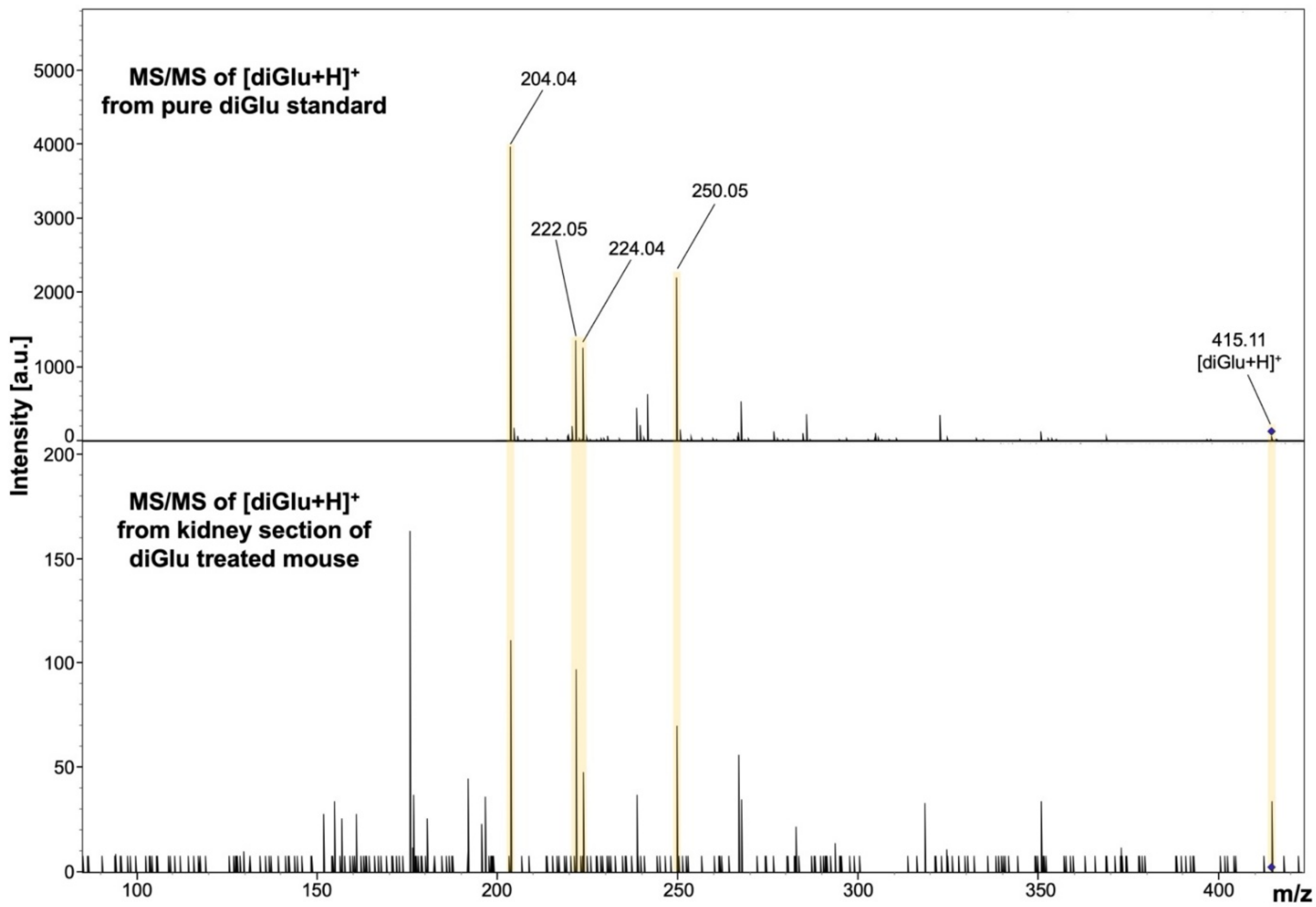

Figure S5: MS/MS fragmentation spectra of $[diGlu+Na]^+$ from pure diGlu standard (top) and from kidney tissue (bottom).

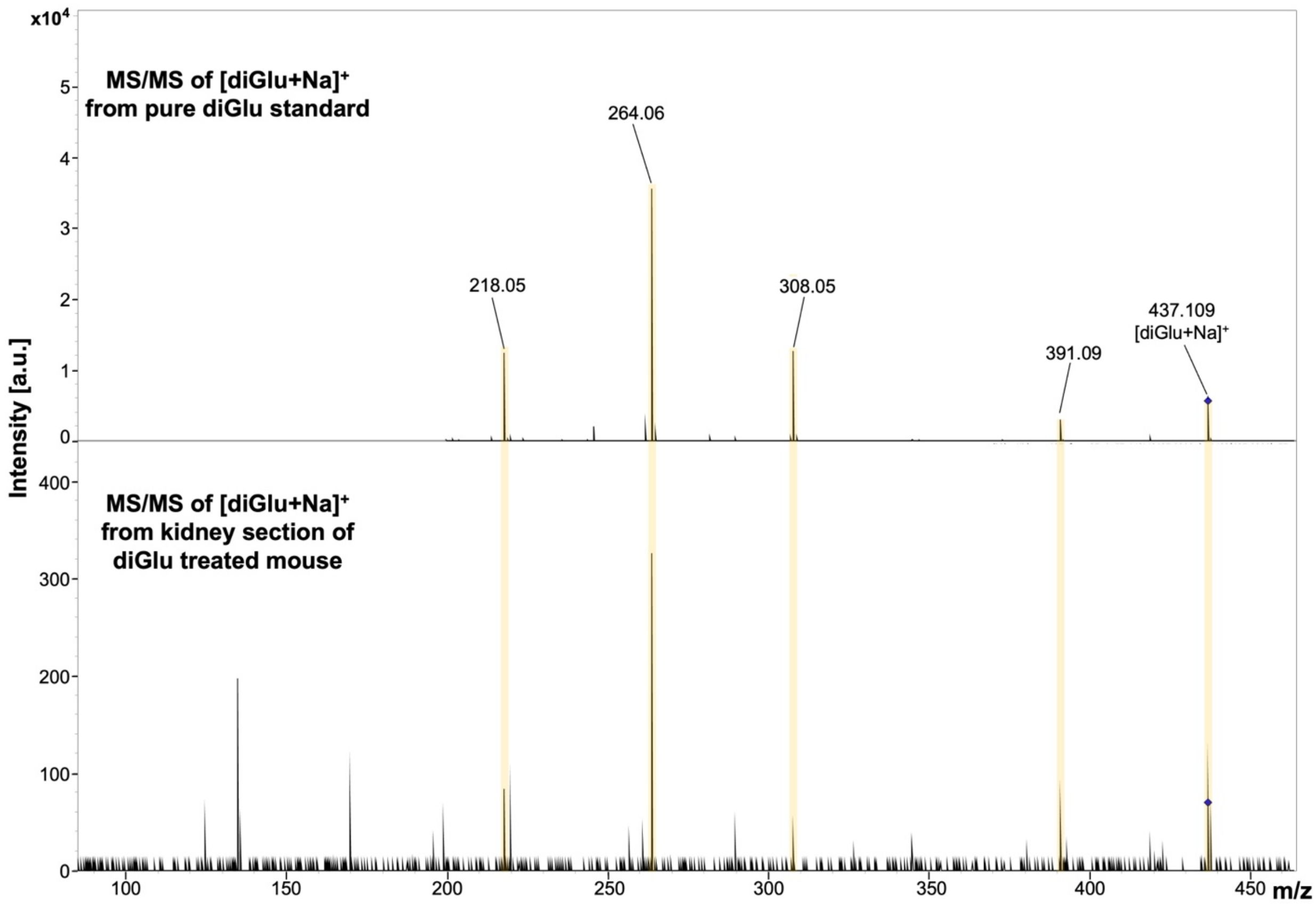

Figure S6: Delayed kidney H&E staining 48 h after injection in control, iopamidol, and I45DC-diGlu groups.

Representative H&E-stained kidney sections from control mice, mice treated with iopamidol, and mice treated with I45DC-diGlu, collected 48 h after injection. Images are shown for cortex and outer medulla at 10× and 20× magnification. Renal architecture remained preserved across groups, with no overt histopathological abnormalities observed at this delayed time point.

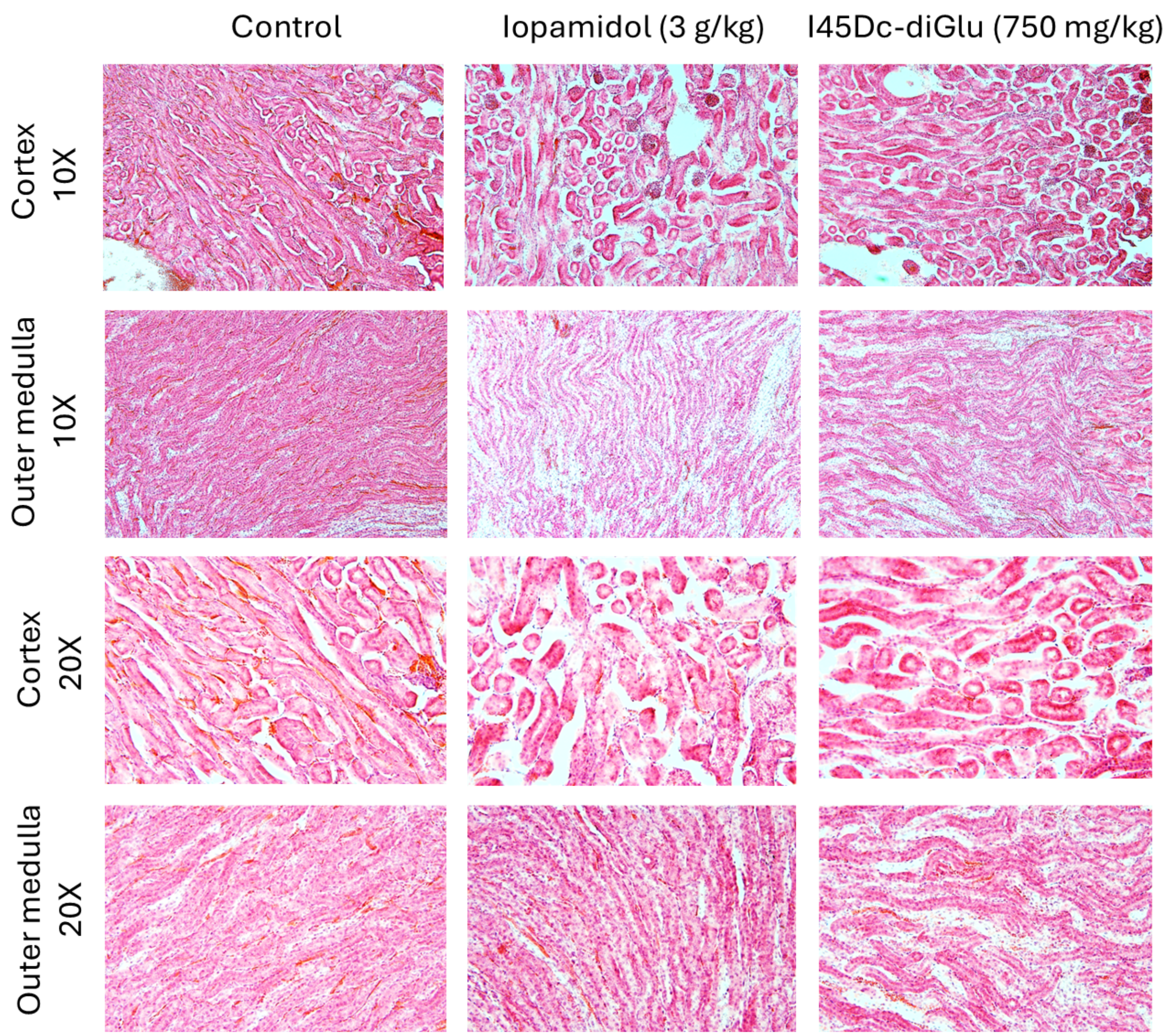

In Vitro MRI Data Processing and pH Calibration

To evaluate the pH sensitivity of I45DC-diGlu, a series of phantoms were prepared in phosphate-buffered saline (PBS) at physiological pH values ranging from 5.7 to 7.1. CEST MRI scans were acquired on an 11.7 T MRI scanner using continuous wave saturation at multiple frequency offsets. The resulting Z-spectra (**Figure S7a**) revealed two distinct saturation dips at 4.5 ppm and 7.7 ppm corresponding to the exchangeable amide protons of I45DC-diGlu. MTR_{asym} analysis (**Figure S7b**) demonstrated strong pH dependence of CEST contrast at these offsets, with higher pH values showing increased MTR amplitude due to faster proton exchange. To quantify pH, a ratiometric approach was employed, calculating the saturation transfer (ST) ratio as the signal amplitude at 4.5 ppm divided by that at 7.7 ppm. A calibration curve was generated by fitting ST ratios against electrode-measured pH values using a third-order polynomial (**Figure S7c**).

This model enabled the conversion of CEST signal ratios into quantitative pH values. Validation using phantom pH maps (**Figure S7d**) confirmed excellent agreement between MRI-derived and true pH values, demonstrating the high accuracy and spatial resolution of I45DC-diGlu for pH mapping under physiological conditions.

Calibration curves were acquired for a range of saturation powers (B_1 = 2.0 to 8.0 μT) (**Figure S8a–f**). The pH-dependent changes in ST ratio demonstrated clear, non-linear relationships that were strongly dependent on B_1 power. Among these, B_1 = 5.0 μT yielded the optimal balance between sensitivity and accuracy across the physiological pH range. Validation against electrode-based measurements confirmed the fidelity of the CEST-derived pH values, with a root mean square error (RMSE) of 0.102 (**Figure S8g**). These results establish a reliable and reproducible framework for in vivo pH quantification using I45DC-diGlu and highlight the importance of B_1 optimization in ratiometric CEST MRI analysis.

Figure S7. In vitro validation of I45DC-diGlu for ratiometric CEST MRI-based pH mapping

(a) Normalized Z-spectra of I45DC-diGlu phantoms at pH values ranging from 5.7 to 7.1 and a control sample (PBS without contrast agent), showing saturation dips at 4.5 and 7.7 ppm due to exchangeable protons.
(b) Corresponding MTR_{asym} plots demonstrate pH-dependent changes in signal amplitude at 4.5 and 7.7 ppm, enabling ratiometric contrast generation.
(c) Calibration curve generated by fitting a polynomial function to the saturation transfer (ST) ratio (4.5 ppm / 7.7 ppm) versus pH data points.
(d) CEST MRI pH map of 7 phantoms overlaid on CEST image, confirming accurate spatial localization and quantification of pH values between 5.7 and 7.1. The color scale reflects the estimated pH, demonstrating the utility of I45DC-diGlu for sensitive and precise pH mapping in vitro.

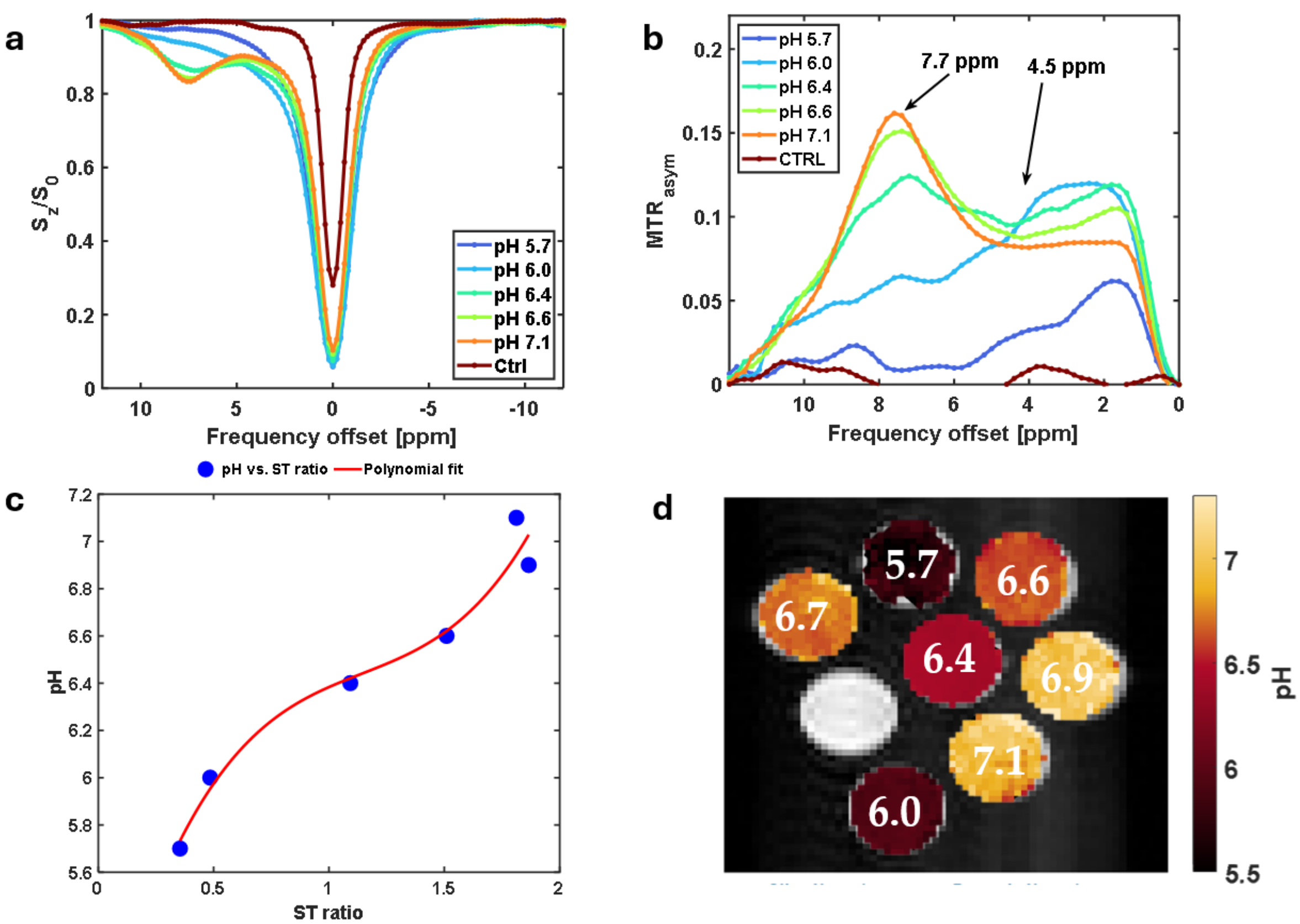

Figure S8. B_1-dependence of ratiometric pH calibration for I45DC-diGlu and validation of CEST MRI accuracy.

(a–f) Polynomial calibration curves relating pH to the saturation transfer (ST) ratio (4.5 ppm / 7.7 ppm) for I45DC-diGlu phantoms imaged under varying saturation powers: **(a)** B_1 = 2.0 μT, **(b)** 3.0 μT, **(c)** 4.0 μT, **(d)** 5.0 μT, **(e)** 6.0 μT, **(f)** 8.0 μT. Blue dots indicate experimental data; red lines show polynomial fits. The ST ratio-pH relationship improves in monotonicity and fit quality between B_1 = 4.0–6.0 μT, with B_1 = 5.0 μT selected for subsequent studies.
(g) Validation plot comparing electrode-measured pH values (x-axis) to CEST-derived pH values (y-axis) at B_1 = 5.0 μT. Blue bars indicate mean ± SD for each phantom; red line represents the linear regression fit, yielding a root mean square error (RMSE) of 0.102.

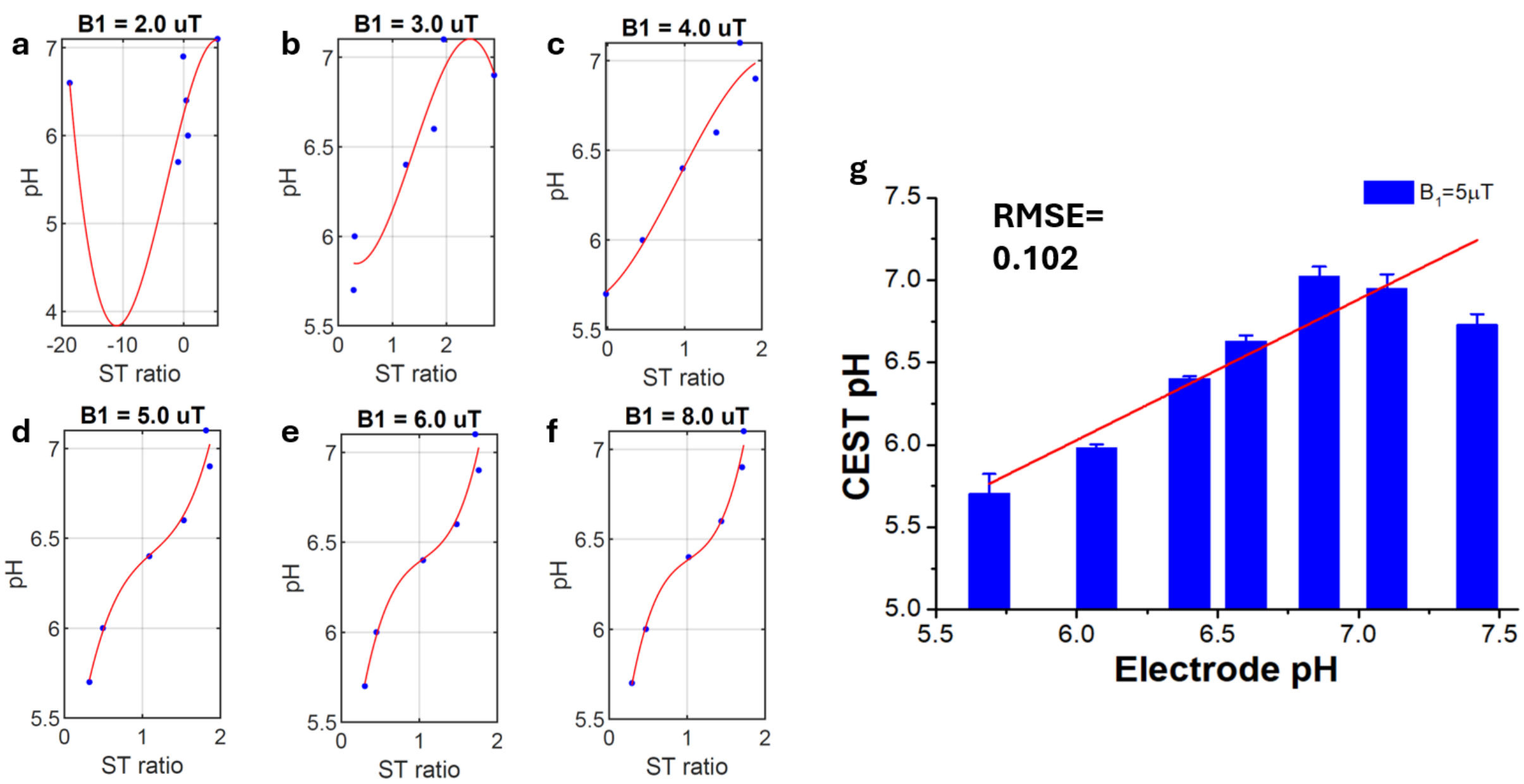

In vivo MRI experiment workflow

Figure S9 summarizes the in vivo CEST MRI protocol used to assess renal pH and clearance kinetics of I45DC-diGlu in 3-4-month-old male C57BL/6J mice. The imaging workflow begins with anatomical localization using high-resolution T_2-weighted scans, followed by WASSR acquisition for B_0 field correction. The core of the protocol involves dynamic CEST MRI using frequency-selective saturation at 4.5 ppm and 7.7 ppm, targeting the two exchangeable proton pools of I45DC-diGlu. The acquisition sequence comprises 6 baseline M_0 images, followed by 179 frequency-saturated images (capturing both 4.5 ppm and 7.7 ppm offsets), and concludes with 6 post-injection M_0 images.

Representative CEST-weighted images collected across timepoints illustrate the evolution of renal signal during I45DC-diGlu uptake and clearance. Image datasets were processed using a custom graphical user interface (GUI) designed for CEST analysis. The GUI enabled B_0 correction, saturation transfer (ST) ratio analysis, dynamic signal curve fitting, and pH map reconstruction. This streamlined imaging and data processing pipeline allowed for efficient, high-resolution, and reproducible functional mapping of the kidneys in vivo, and formed the foundation for quantitative assessment of renal physiology and contrast agent pharmacokinetics using I45DC-diGlu.

Figure S9: Schematics of the in vivo CEST MRI protocol for renal imaging using I45DC-diGlu in mice and CEST MRI acquisition sequence used for dual-offset renal imaging

A) Schematic showing the imaging and analysis pipeline used in 3-4-month-old male C57BL/6J mice. The protocol includes T_2-weighted and WASSR scans for localization and B_0 correction, followed by dynamic CEST imaging (4.5 and 7.7 ppm) with M_0 references acquired pre- and post-injection. Data were processed using a custom GUI for B_0 correction, ST ratio analysis, and pH map generation.

B) Schematic timing diagram of the CEST MRI acquisition used for dual-offset renal imaging.

A

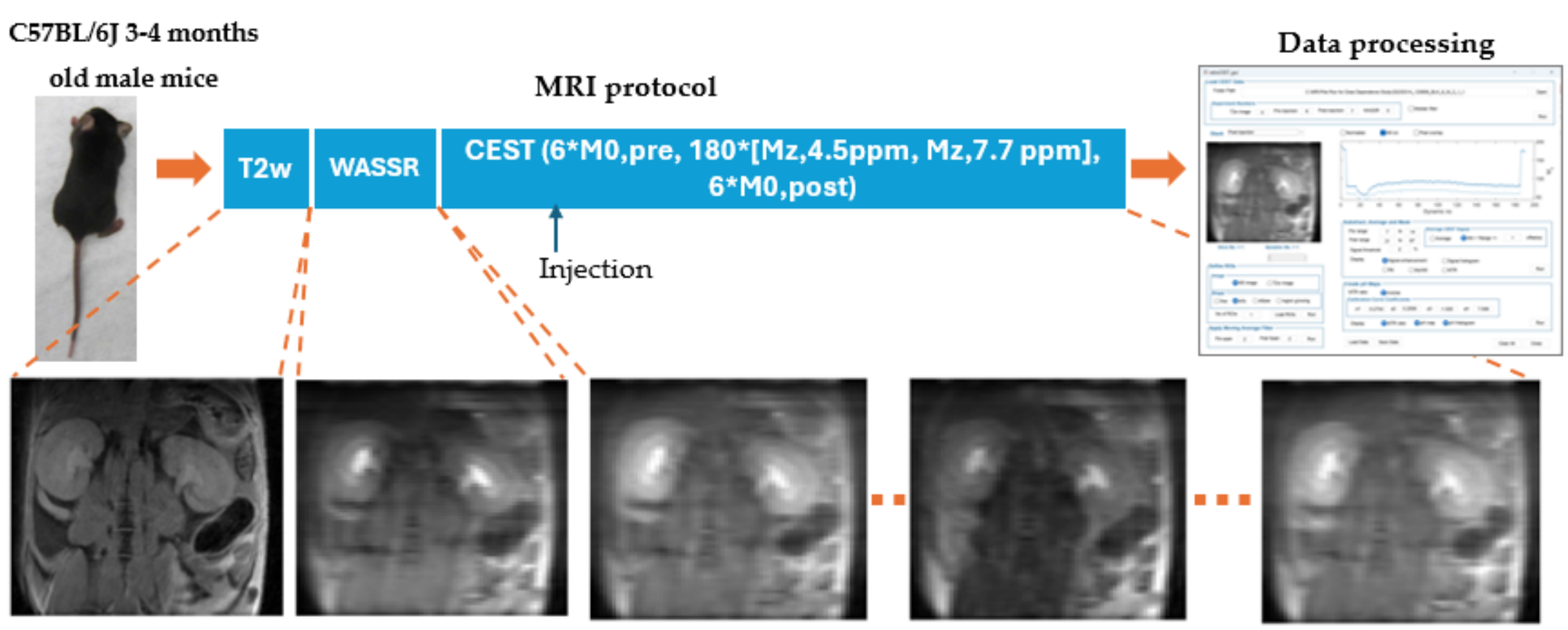

B

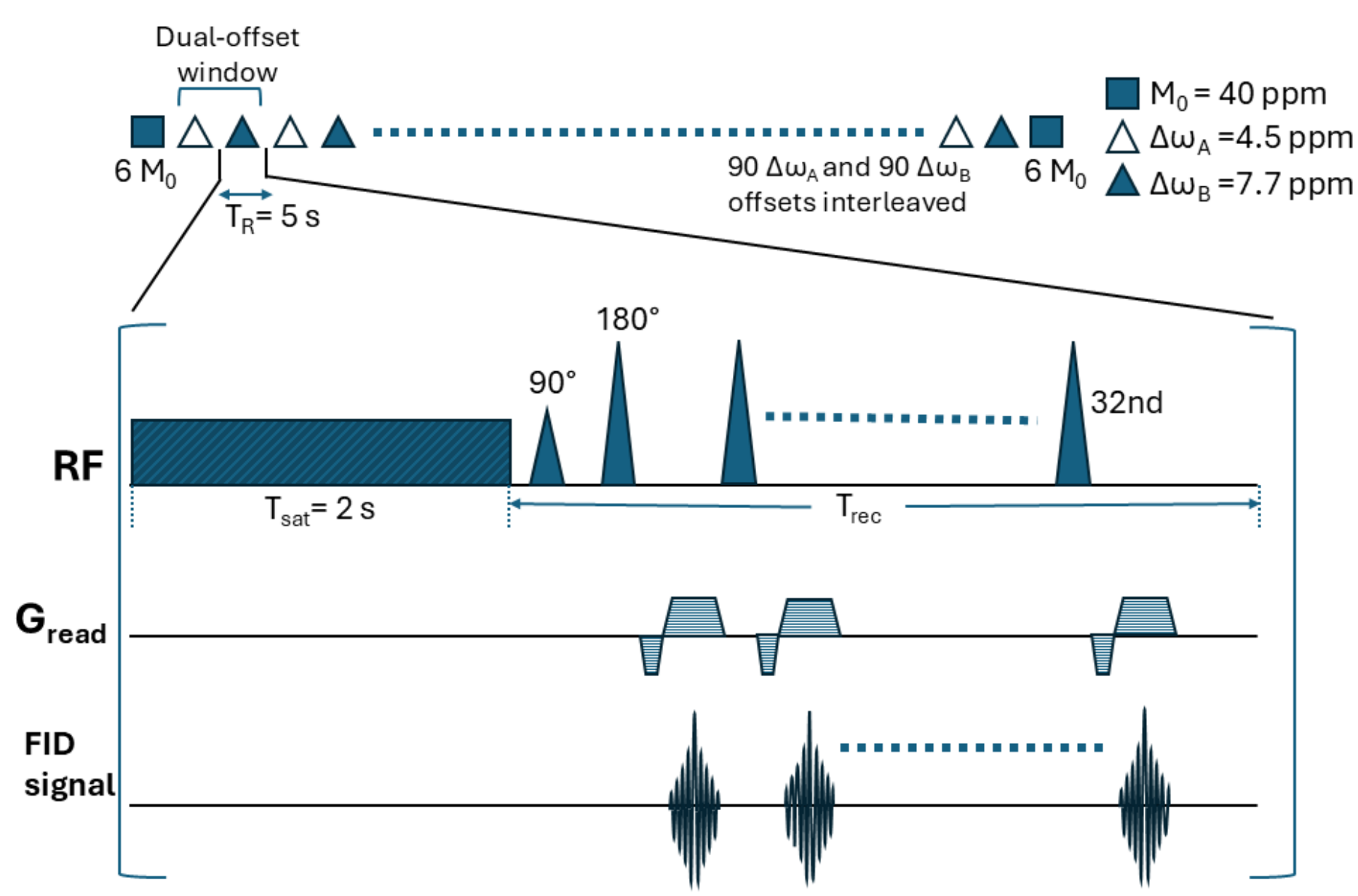

Figure S10. Renal CEST performance of iopamidol and comparison with I45DC-diGlu at under identical experimental conditions.

A) Representative T2-weighted MR image of the kidney. **B)** Corresponding CEST MR image with manually defined regions of interest (ROIs) for cortex (C), outer medulla (OM), and inner medulla (IM). **C,D)** Post-contrast MTR maps acquired at 4.20 ppm and 5.50 ppm, respectively. E,F) Corresponding ΔMTR maps at 4.20 ppm and 5.50 ppm. **G,H)** Regional saturation enhancement (SE) time courses at 4.20 ppm and 5.50 ppm for cortex, outer medulla, and inner medulla. I) MTRratio map derived from the dual-offset analysis. **J)** Corresponding pH map generated from the ratiometric calibration. **K)** Histograms showing regional pH distributions in cortex, outer medulla, and inner medulla. **L)** Comparison of peak saturation enhancement (SEmax) between iopamidol (4.2 ppm) and I45DC-diGlu (7.7 ppm), showing no significant difference(ns). The iopamidol dose (1.5 g iodine/kg) was selected based on prior studies to provide a peak saturation enhancement comparable to that achieved with the highest I45DC-diGlu dose used in this study (750 mg/kg).

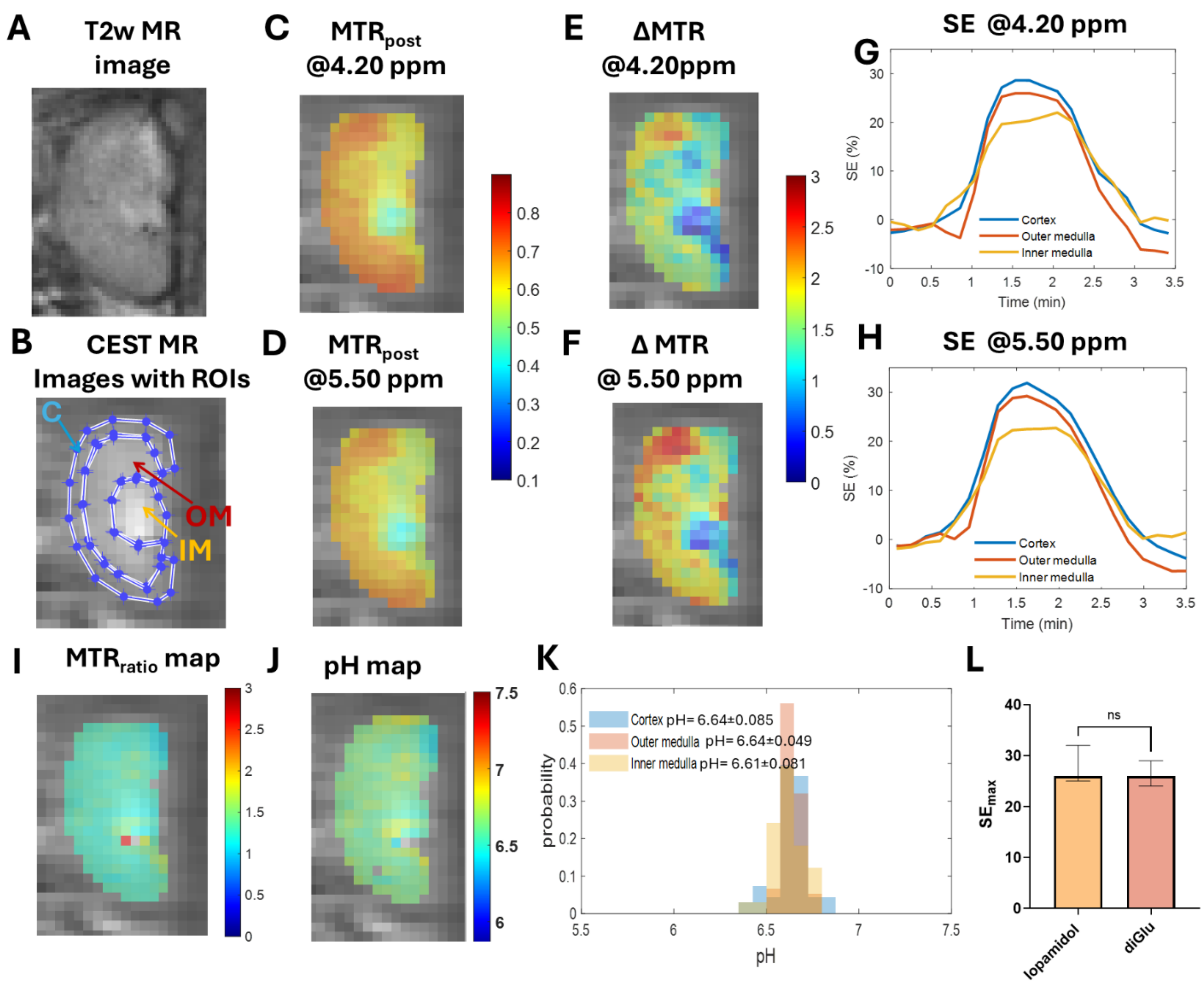

Figure S11. Summary of dose- and region-dependent CEST-derived pH and pharmacokinetic metrics for I45DC-diGlu in mouse kidney

Summary of regional CEST-derived pH and pharmacokinetic parameters across dose levels following intravenous administration of I45DC-diGlu. (A) CEST-derived pH measured in inner medulla (IM), outer medulla (OM), and cortex (C) at 250, 500, and 750 mg kg^{-1}, showing relatively stable regional pH values across doses. (B) Peak signal enhancement at 7.7 ppm (SE_{max}) increases with dose, with the strongest enhancement observed in the outer medulla. (C) Time-to-peak (TTP) shows a modest dose-dependent increase. (D) Half-life ($t_{1/2}$) increases with dose, indicating more prolonged retention at higher administered levels. (E) Area under the curve (AUC, %·min) increases strongly with dose and is greatest in the outer medulla, consistent with greater cumulative exposure in this region. Bars represent mean ± SD, and overlaid points indicate individual animals. Statistical significance is shown as indicated (*$p < 0.05$, **$p < 0.01$, ***$p < 0.001$, ****$p < 0.0001$; ns, not significant). Abbreviations: IM, inner medulla; OM, outer medulla; C, cortex; SE_{max}, maximum signal enhancement; TTP, time-to-peak; AUC, area under the curve.

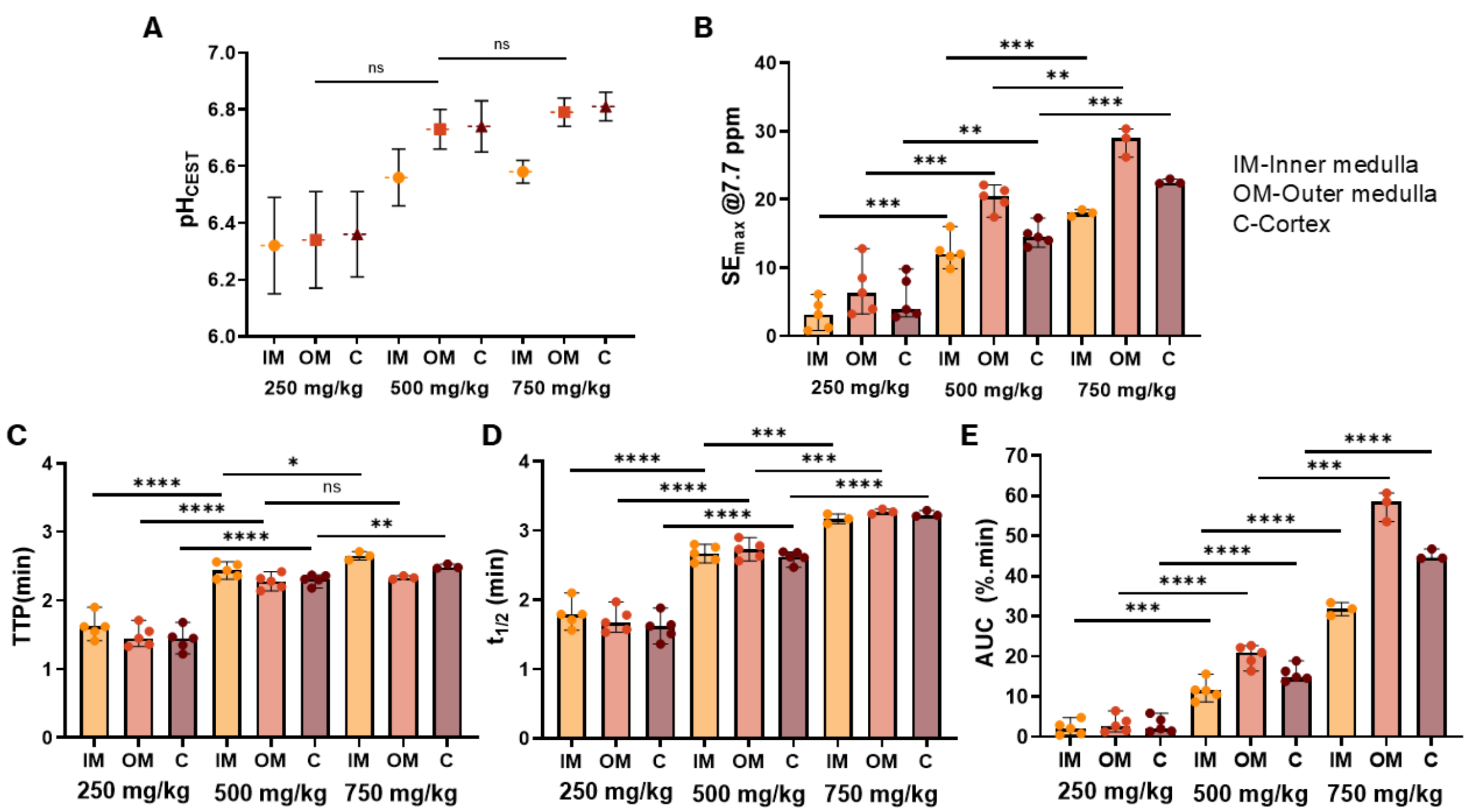

Figure S12: Residual diagnostics for kinetic model fits across dose and renal regions. Residuals (data − fit; SE%) versus time are shown for inner medulla, outer medulla, and cortex at 250, 500, and 750 mg/kg. For each ROI, markers depict residuals from the log-normal (LN, ×) and Uptake–Plateau–Decay (UPD) fits performed on the original, non-interpolated samples; the dotted line denotes zero residual. At 250–500 mg/kg, residuals are tightly centered around zero (typically within ±2–3 SE%), indicating good descriptive adequacy of both models. At 750 mg/kg, residual variance increases and mild temporal structure appears with the LN model, whereas UPD residuals remain more homoscedastic and closer to zero-consistent with the AICc-based preference for UPD at the highest dose (see main figure). Abbreviations: SE, signal enhancement; LN, log-normal; UPD, Uptake–Plateau–Decay.

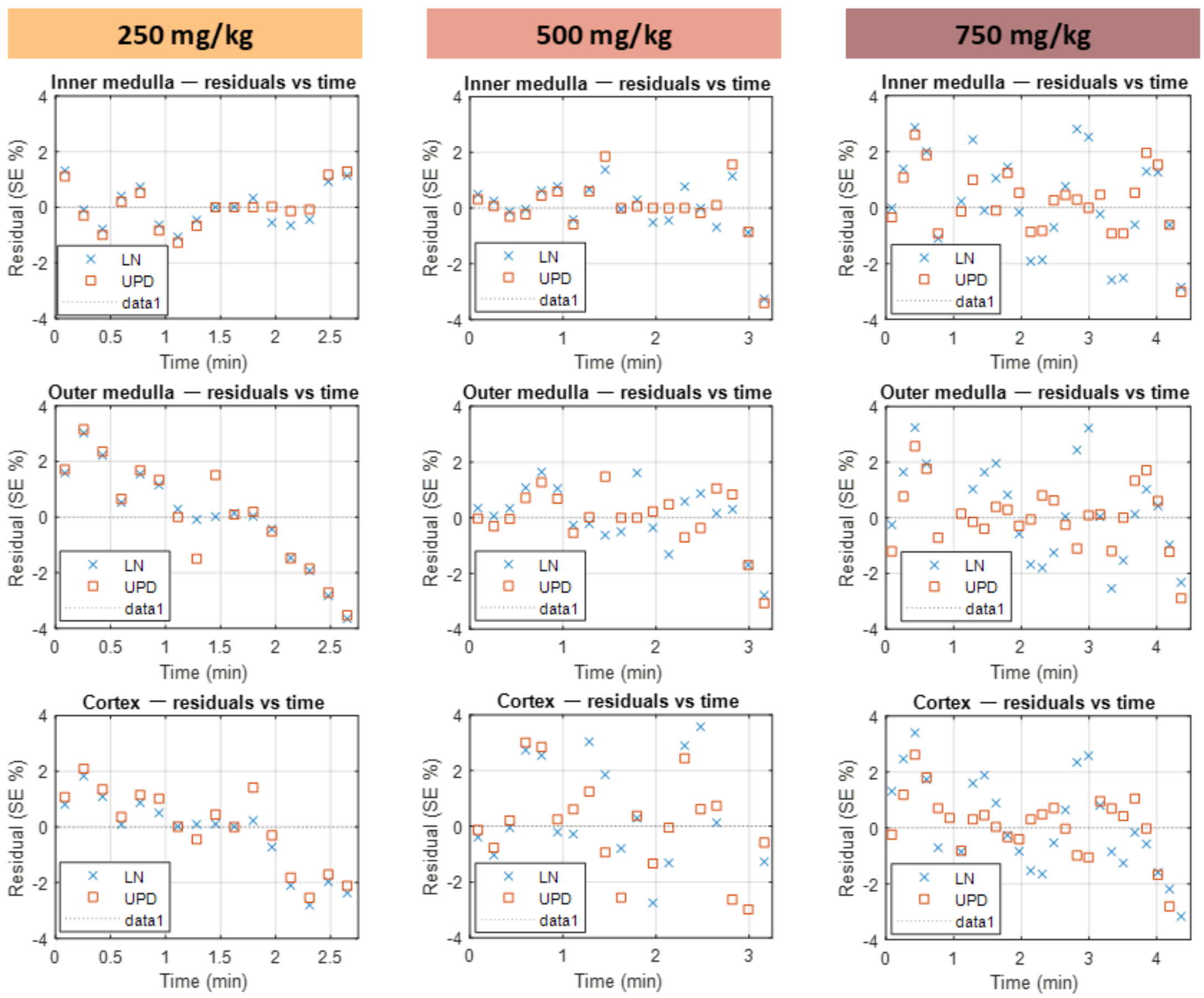

Figure S13: Residual distributions (PDF-normalized histograms) for kinetic model fits.

For each renal region (inner medulla, outer medulla, cortex) and dose (250, 500, 750 mg/kg), histograms show the probability density of residuals (data − fit; SE%) from the log-normal (LN) and Uptake–Plateau–Decay (UPD) models fitted to the original, non-interpolated samples. Distributions centered near zero and with narrow spread indicate good fit quality. At 250–500 mg/kg, both models yield tight, near-symmetric residuals; at 750 mg/kg, UPD residuals are generally more compact and closer to zero than LN, consistent with the AICc-based model preference reported in the main figure. Abbreviations: SE, signal enhancement; LN, log-normal; UPD, Uptake–Plateau–Decay.

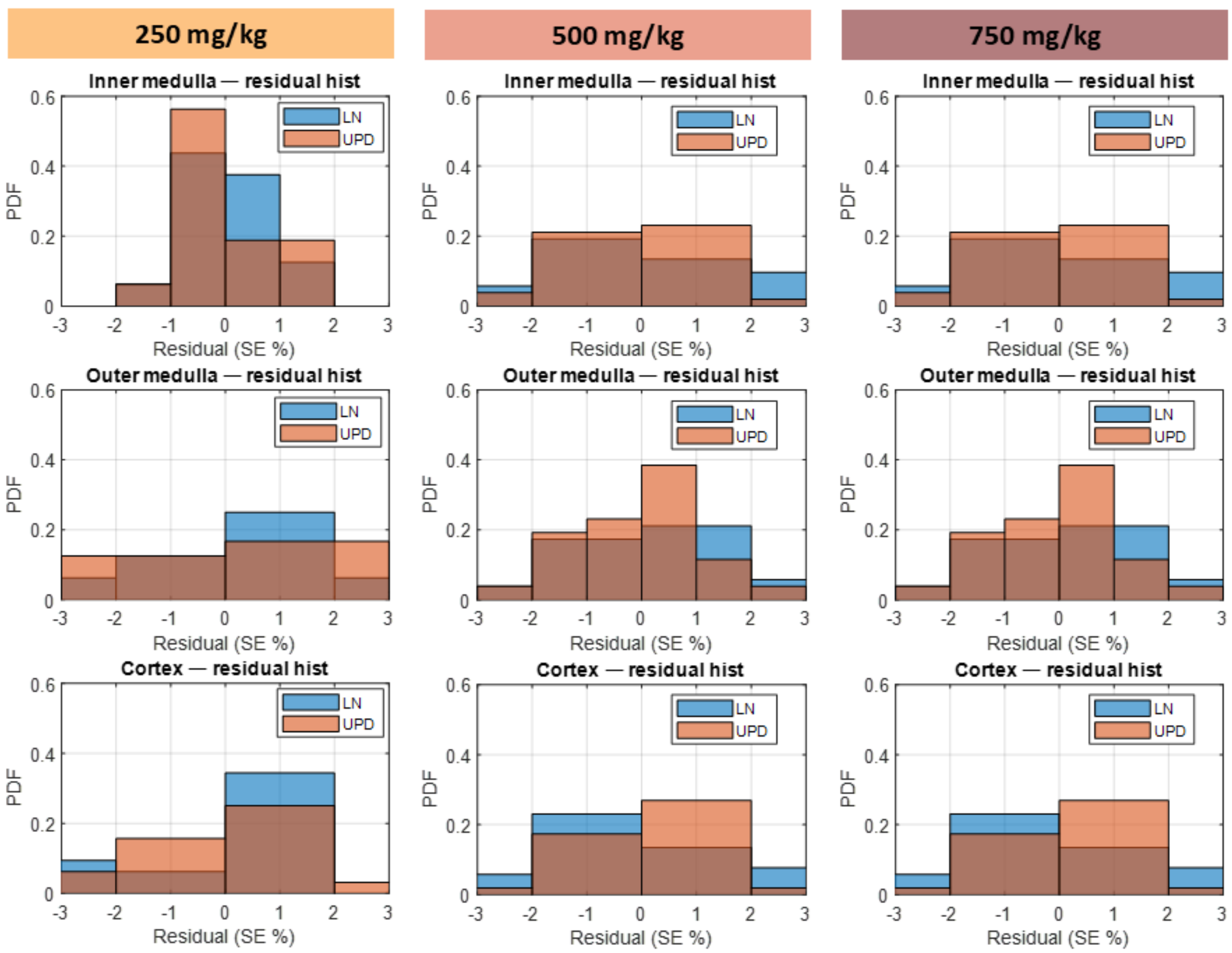

Figure S14. Residual frequency distributions and residual autocorrelation analysis across renal compartments.

The top row shows residual frequency distributions for the log-normal (LN) and uptake-plateau-decay (UPD) models across cortex, outer medulla, and inner medulla for dosage of 750mg kg^{-1}. The bottom row shows autocorrelation function (ACF) plots of residuals, with lag-1 autocorrelation coefficients (ACF_1) indicated. Dashed lines represent approximate confidence limits. These analyses were used to evaluate residual distribution and temporal dependence as part of model robustness assessment.

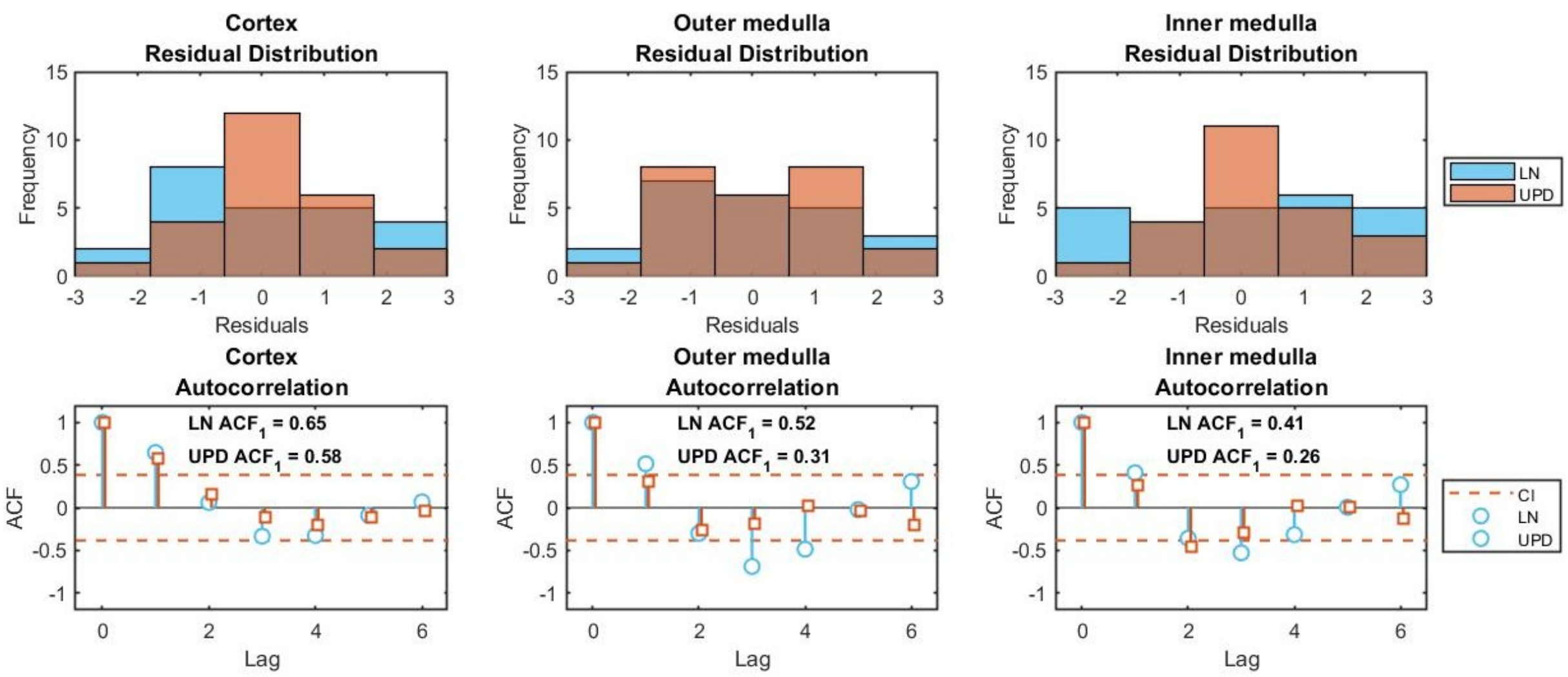

Figure S15. Simulations of ratiometric I45DC-diGlu CEST contrast for 11.7 T and 3T scanners.

(Top) Simulated Z-spectra, MTR_{asym}, and pH-versus-ST_{ratio} calibration curves for I45DC-diGlu at 11.7 T using B_1 = 5 µT, Tsat = 2 s, matching the preclinical acquisition parameters used in this study.

(Bottom) Corresponding simulations at 3 T using B_1 = 2.4 µT saturation, Tsat = 3 s, representing the contrast that could be obtained using body coils for transmission. Although the absolute CEST contrast obtained is lower than in the top panel, the ST_{ratio} remained monotonic across the physiologic pH range and can still be fit by a third order polynomial. These simulations suggest that detection is feasible on 3 T scanners with field-specific optimization of saturation parameters.

Exchange rates used for simulation for the pH values,

$k_{7.7}$ = [35000 14250 6200 4750 4050 3950];

$k_{4.5}$ = [4500 6000 8000 13000 14000 14500];

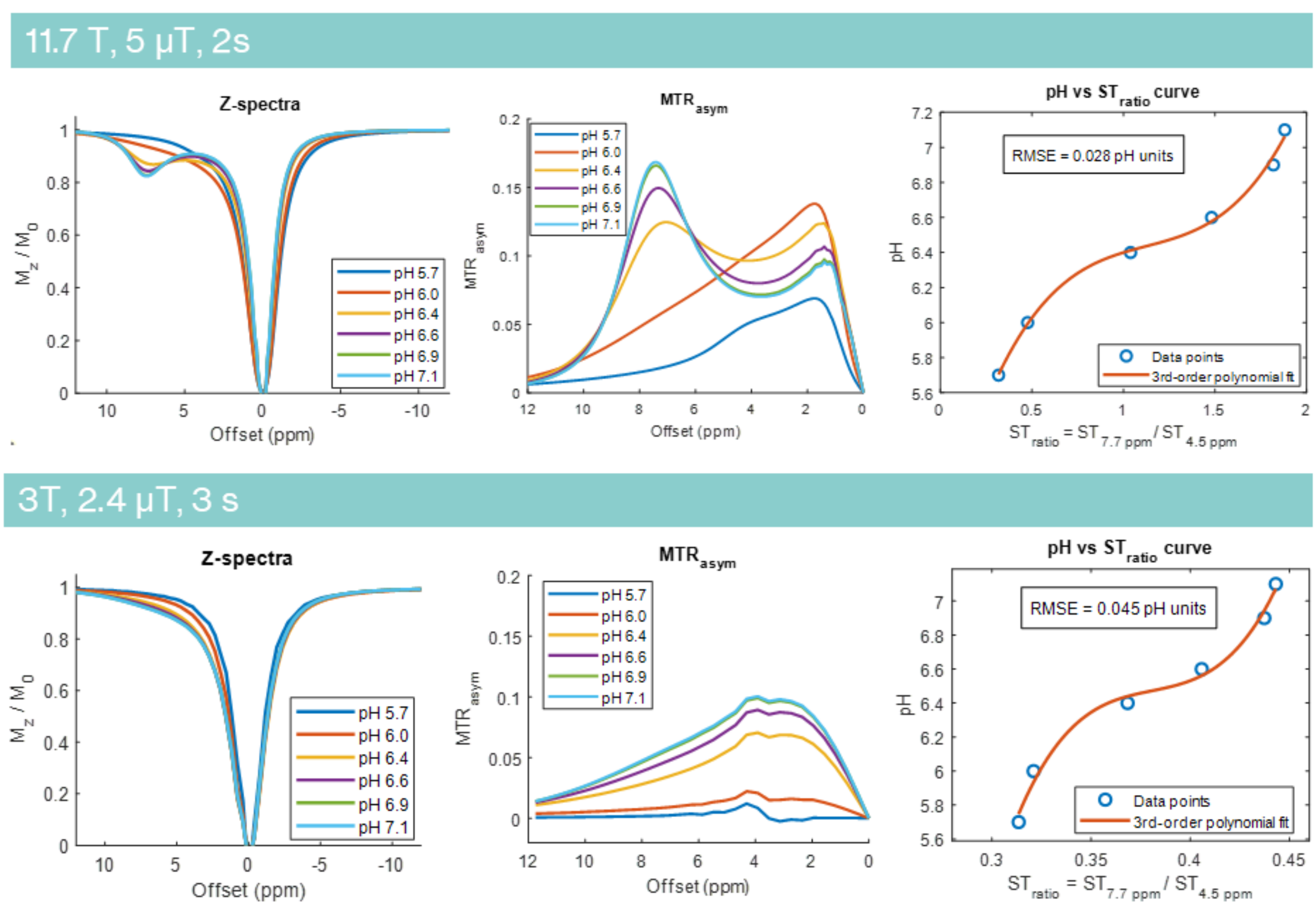

Table S1. ROI-wise PK modeling summary across doses. For representative mice, per-ROI signal-enhancement (SE, %) time-courses at 7.70 ppm were fit with two candidate models-Log-normal (LN) and Uptake–Plateau–Decay (UPD, piecewise)-and the preferred model was chosen by AICc. Reported metrics include: TTP (time-to-peak, min), SE_{max} (%), $Slope_{\uparrow}$ (early uptake slope, %·min^{-1}), $t_{1/2}$ (first post-peak time at which $SE = 0.5\, SE_{\max}$, min), AUC_{total} (baseline-referenced exposure, %·min), AICc, R^2, and RMSE for each model. $AICc_{best}$ is the minimum AICc for that curve; BestModel indicates the selection (ΔAICc>2 interpreted as meaningfully worse for the non-selected model). Doses are shown by row blocks (250, 500, 750 mg/kg); ROIs are cortex (C), outer medulla (OM), and inner medulla (IM).

**Dosage**	**250 mg/kg**			**500 mg/kg**			**750 mg/kg**		
**ROI**	Inner medulla	Outer medulla	Cortex	Inner medulla	Outer medulla	Cortex	Inner medulla	Outer medulla	Cortex
**TTP**	1.62	1.45	1.45	2.31	2.14	2.31	2.65	2.31	2.48
**SEmax**	3.14	6.33	3.91	9.84	17.42	12.98	18.05	30.33	22.34
**$Slope_{\uparrow}$**	0.95	1.85	1.87	4.28	8.03	5.16	8.65	16.53	12.74
**$t_{1/2}$**	1.79	1.67	1.62	2.53	2.56	2.62	3.17	3.24	3.22
**AUC_{total}**	2.11	2.71	2.03	8.68	16.44	13.84	31.87	60.71	44.52
**$AICc_{LN}$**	4.86	33.56	25.27	13.8	17.58	45.65	45.31	44.91	41.02
**$R2_{LN}$**	0.68	0.65	0.62	0.93	0.97	0.75	0.93	0.97	0.96
**$RMSE_{LN}$**	0.71	1.73	1.34	0.98	1.08	2.26	1.86	1.85	1.71
**$AICc_{UPD}$**	26.3	55.92	46.33	31.15	31.56	49.59	41.39	31.79	40.04
**$R2_{UPD}$**	0.66	0.61	0.6	0.93	0.98	0.87	0.96	0.99	0.98
**$RMSE_{UPD}$**	0.73	1.83	1.36	1.02	1.03	1.66	1.38	1.15	1.35
**$AICc_{best}$**	4.86	33.56	25.27	13.8	17.58	45.65	41.39	31.79	40.04
**Best Model**	Log-normal	Log-normal	Log-normal	Log-normal	Log-normal	Log-normal	UPD (piecewise)	UPD (piecewise)	UPD (piecewise)

Table S2. Post hoc pairwise comparisons of AUC across dose groups with nonparametric and supporting statistical analyses

The mean difference (95% CI) represents the difference between group means with the corresponding 95% confidence interval estimated from Welch's *t*-test, indicating the magnitude and uncertainty of the between-group effect. The median difference represents the difference between group medians for the nonparametric comparison. W denotes the Wilcoxon rank-sum statistic, defined as the sum of ranks for one group after pooling and ranking all observations. The equivalent Mann-Whitney U statistic was derived from W as $U = W - n_1(n_1 + 1)/2$. Exact p-values were obtained from the Wilcoxon rank-sum/Mann-Whitney test and used as the primary inference because of small sample size and non-normal data. Permutation p-values were generated from 10,000 iterations as an assumption-free validation. Welch's t-test p-values are reported as supporting parametric results. Hedges' g was used as the bias-corrected standardized effect size for small samples, with values greater than 0.8 interpreted as large effects. Cohen's d is also reported as an uncorrected standardized effect size for comparison. Observed power was estimated using a two-sample t-test framework based on the observed effect size (Cohen's d), sample size, and α = 0.05, and is reported as a supporting metric.

Comparison	Mean difference (95% CI)	Median difference	Rank-sum statistic (W)	Equivalent U	Exact p-value	Permutation p-value	Welch t-test p-value	Hedges' g	Cohen's d	Observed power
**750 vs 500 mg/kg**	9.33 (4.25 to 14.42)	8	21	15	0.0357	0.0186	0.00935	4	4.6	0.9996
**500 vs 250 mg/kg**	12.60 (8.21 to 16.99)	16	40	25	0.0079	0.0082	0.00047	4.05	4.48	1
